# Mitofusin 1 silencing decreases the senescent associated secretory phenotype, promotes immune cell recruitment and delays melanoma tumor growth after chemotherapy

**DOI:** 10.1038/s41598-024-51427-7

**Published:** 2024-01-09

**Authors:** Doménica Tarallo, Jennyfer Martínez, Alejandro Leyva, Amy Mónaco, Carolina Perroni, Marcos Tassano, Juan Pablo Gambini, Mónica Cappetta, Rosario Durán, María Moreno, Celia Quijano

**Affiliations:** 1 Departamento de Bioquímica, Facultad de Medicina, and Centro de Investigaciones Biomédicas (CEINBIO), Universidad de la República, Montevideo, Uruguay; 2 Institut Pasteur de Montevideo and Instituto de Investigaciones Biológicas Clemente Estable (IIBCE), Montevideo, Uruguay; 3 Departamento de Desarrollo Biotecnológico, Instituto de Higiene, Facultad de Medicina, Universidad de la República, Montevideo, Uruguay; 4 Area Radiofarmacia, Centro de Investigaciones Nucleares, Facultad de Ciencias, Universidad de la República, Montevideo, Uruguay; 5 Centro Uruguayo de Imagenología Molecular (CUDIM) and Centro de Medicina Nuclear (CMN), Hospital de Clínicas Dr. Manuel Quintela, Facultad de Medicina, Universidad de la República, Montevideo, Uruguay; 6 Departamento de Genética, Facultad de Medicina, Universidad de la República, Montevideo, Uruguay

**Keywords:** Senescence, Mitochondria, Melanoma

## Abstract

Cellular senescence is a therapy endpoint in melanoma, and the senescence-associated secretory phenotype (SASP) can affect tumor growth and microenvironment, influencing treatment outcomes. Metabolic interventions can modulate the SASP, and mitochondrial energy metabolism supports resistance to therapy in melanoma. In a previous report we showed that senescence, induced by the DNA methylating agent temozolomide, increased the level of fusion proteins mitofusin 1 and 2 in melanoma, and silencing *Mfn1* or *Mfn2* expression reduced interleukin-6 secretion by senescent cells. Here we expanded these observations evaluating the secretome of senescent melanoma cells using shotgun proteomics, and explored the impact of silencing *Mfn1* on the SASP. A significant increase in proteins reported to reduce the immune response towards the tumor was found in the media of senescent cells. The secretion of several of these immunomodulatory proteins was affected by *Mfn1* silencing, among them was galectin-9. In agreement, tumors lacking mitofusin 1 responded better to treatment with the methylating agent dacarbazine, tumor size was reduced and a higher immune cell infiltration was detected in the tumor. Our results highlight mitochondrial dynamic proteins as potential pharmacological targets to modulate the SASP in the context of melanoma treatment.

## Introduction

Senescent cells are secretory cells that can be found in several physiological and pathological conditions, including cancer. Cellular senescence is induced by stress stimuli, such as DNA damage, oncogene activation or loss of tumor suppressors^1,2^. This cellular state involves proliferation arrest mediated by the cyclin kinase inhibitors p21 and/or p16, changes in morphology and chromatin, apoptosis resistance, and increase in lysosome content, among others^1,2^. Senescent cells also synthesize and export multiple protein factors by both conventional and unconventional pathways, known as the senescence associated secretory phenotype (SASP), exerting an important influence on their microenvironment^3,4^.

In cancer, chemotherapy, radiotherapy and targeted therapies can induce senescence, and the SASP can generate a either a tumor suppressing or tumor promoting microenvironment affecting invasion, therapy resistance and evasion of the immune system^5–9^. These observations highlight the need of a thorough characterization of the factors secreted by senescent cells, as well as the signaling pathways and mechanisms that support the SASP.

Our group and others have shown that mitochondria play a relevant role in protein secretion in senescent cells^10–12^. In a recent report we observed that senescent melanoma cells, generated after the exposure to the DNA-methylating chemotherapeutic temozolomide (TMZ)^13^, undergo profound changes in mitochondrial dynamics^10^. We observed an important increase in mitofusin 1 and 2 protein levels that was required for the secretion of interleukin 6 (IL-6), a component of the SASP^10^. Mitofusin 1 and 2 are GTPases required for mitochondrial fusion, they are embedded in the mitochondrial external membrane where they form oligomers linking different mitochondria^14^. These proteins are also required for mitochondrial DNA (mtDNA) maintenance^15^, and tether mitochondria to other organelles and cellular structures such as the endoplasmic reticulum (ER) and peroxisomes^16–18^. Mitofusin 2 has also been reported to bind mitochondria to the nucleus^19^ and to the Miro/Milton mitochondrial transport complex^20^. Homozygous knockout animals for either of these proteins are not viable, and silencing *Mfn1* or *Mfn2* strongly impacts on mitochondrial metabolism, autophagy and overall cell physiology^15,21,22^.

Here we study the impact of silencing *Mfn1* on the secretome of senescent and non-senescent melanoma cells, using shotgun proteomics. Our results show that *Mfn1* silencing reduced the levels of several extracellular proteins secreted by both senescent and non-senescent cells, many of which are reported to present immunomodulatory properties. We validated the results obtained for galectin-9, a glycoprotein of the galectin family encoded by the *Lgals9* gene, recently identified as an immunosuppressive factor in cancer. Lack of mitofusin 1 reduced galectin-9 protein levels and strongly influenced immune cell recruitment and tumor development, highlighting the potential of mitochondria as a target in cancer treatment.

## Results

### *Mfn1* silencing in senescent and non-senescent melanoma cells

**Figure 1:**
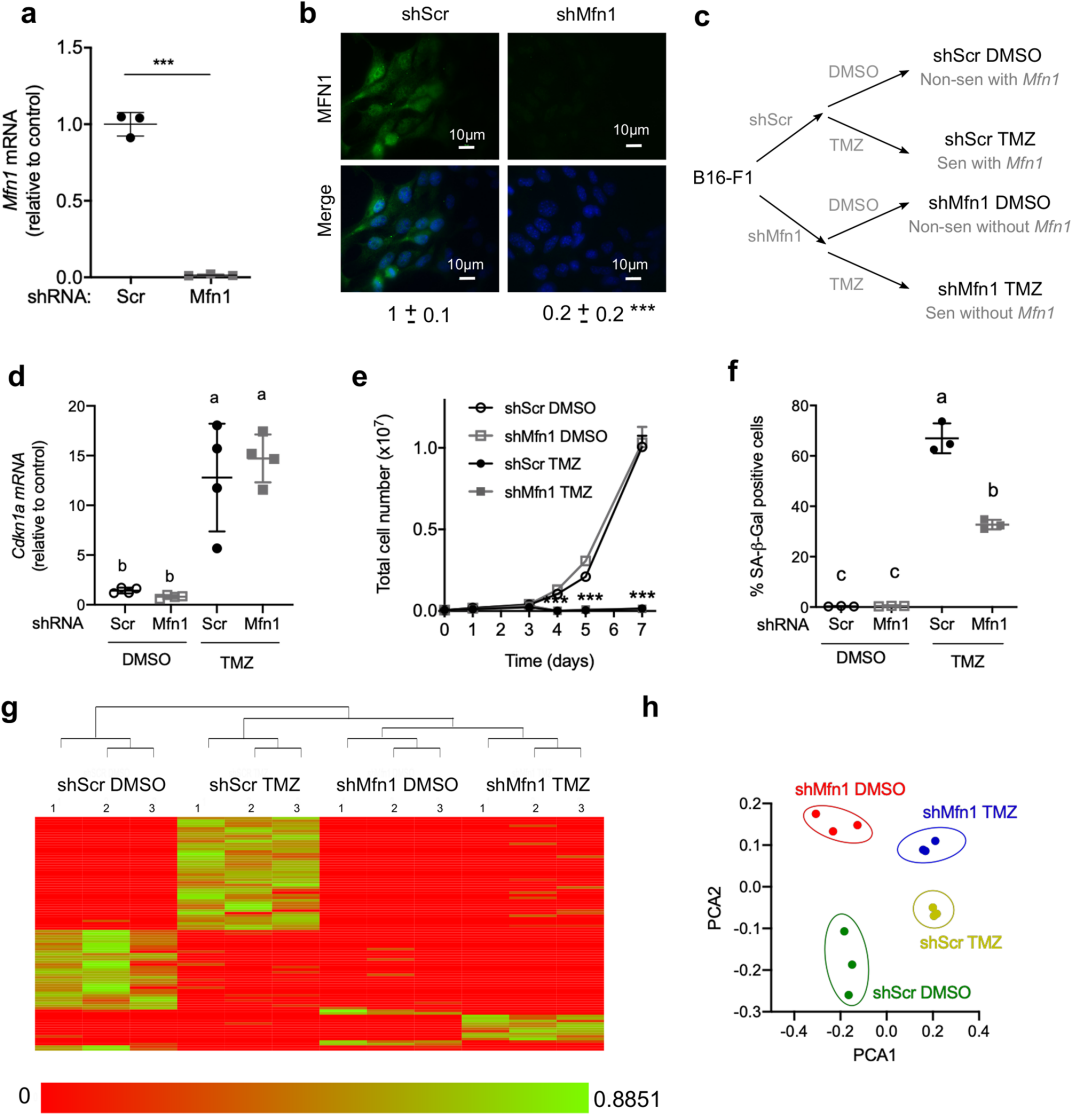
*Mfn1* silencing in melanoma cells affects the senescence associated secretory phenotype (SASP). B16-F1 cells were transduced with lentiviral particles carrying shScr or shMfn1 and selected with puromycin. (**a**) *Mfn1* expression was determined by RT-qPCR and reported relative to control value. Unpaired t-test, two-tails, ***P < 0.0001. (**b**) Representative images of mitofusin 1 (MFN1) protein levels, assessed by immunocytochemistry. Unpaired t-test, two-tails, ***P < 0.0001 (n = 3). (**c**) ShScr and shMfn1 cells were then exposed twice to TMZ (200 μM) or the vehicle DMSO for five hours with a 24 h interval. Samples were analyzed five days after the last exposure to the drug. A schematic representation of the different groups obtained after transduction and treatment with TMZ or vehicle is shown. (**d**) mRNA levels of *Cdkn1a* (p21). Two-way ANOVA, main effects of treatment (P < 0.0001), shRNA (P = 0.67) and their interaction (P = 0.41). Tukey post-hoc for multiple comparisons, different letters are significantly different (P ≤ 0.0008). (**e**) Number of live cells in the culture assessed with Trypan blue at different time points after the last treatment. Two-way ANOVA and Tukey post-hoc for multiple comparisons, ***P < 0.0001 ShScr TMZ vs ShScr DMSO, and ShMfn1TMZ vs ShScr DMSO. (**f**) SA-β-galactosidase activity. The percentage of positive cells is shown. Two-way ANOVA, main effects of treatment (P < 0.0001), shRNA (P < 0.0001) and their interaction (P < 0.0001). Tukey post-hoc for multiple comparisons, different letters are significantly different (P < 0. 0001). In (**a**,**b**,**d**,**e**,**f**) results are the mean ± SD (n = 3–4). (**g**) Conditioned media was obtained as described in Methods. Protein content was analyzed by shotgun proteomics, and a heatmap generated with the Clustergram module from PatternLab for Proteomics software. (**h**) Principal components analysis (PCA) was performed with the Buzios module from PatternLab for Proteomics software.

In order to study the role of mitofusin 1 in protein secretion in senescence we worked with a stable cell line obtained by transducing mouse B16-F1 melanoma cells with lentiviral particles carrying shRNA targeting Mfn1 (shMfn1), or a scrambled sequence (shScr), and selected with puromycin^10^. Through this procedure we obtained cells in which *Mfn1* expression (Fig. 1a) and mitofusin 1 protein were significantly lower than in control (shScr) cells (Fig. 1b).

Both cell lines were exposed to DMSO (vehicle) or the methylating agent temozolomide (TMZ), as described previously^10^, generating control non-senescent cells (shScr DMSO) and senescent cells (shScr TMZ); as well as non-senescent and senescent cells without mitofusin 1 (shMfn1 DMSO and shMfn1 TMZ, respectively) (Fig. 1c). Exposure of melanoma cells to TMZ (200 μM) increased *Cdkn1a* (p21) expression (Fig. 1d), inhibited culture growth (Fig. 1e), and increased SA-β-galactosidase (SA-β-Gal) staining (Fig. 1f), as previously reported by our group^10^. *Mfn1* silencing did not affect the sensitivity of melanoma cells to the drug, since similar half-maximal inhibitory concentrations (IC50) were found for cells transfected with shMfn1 (69 ± 4 μM) or shScr (66 ± 2 μM) (Supplementary Fig. S1 online). Lack of mitofusin1 did not affect *Mfn2* expression in non-senescent (1.0 ± 0.3 in shScr DMSO versus 1.2 ± 0.7 in shMfn1 DMSO cells P = 0.7, n = 3–4) or senescent cells (2.6 ± 0.7 in shScr TMZ versus 1.9 ± 0.6 in shMfn1 TMZ cells P = 0.2, n = 3–4). Lack of mitofusin 1 did not reduce *Cdkn1a* (Fig. 1d) expression or prevent proliferation arrest (Fig. 1e), but the percentage of SA-β-Gal positive cells was lower (Fig. 1f).

Since TMZ had been reported to affect mtDNA integrity^23,24^, we measured mtDNA damage in the D-loop, which is considered particularly sensitive to genotoxic agents^25,26^. A low number of lesions, fewer than those generated by exposure to the oxidant H_2_O_2_ (1.5 ± 0.6 versus 5.4 ± 0.3 lesions/10 kb, P = 0.0006), was detected in senescent cells (Supplementary Fig. S2 online). We also measured the mtDNA/nDNA ratio in senescent cells, and it presented a threefold increase with respect to control cells (Supplementary Fig. S2 online). Finally, to check if DNA damage affected mtDNA transcription we evaluated the expression of mitochondrial genes encoding subunits of the different Complexes I-V and rRNA 12S. Treatment with TMZ did not reduce the expression of any of the assessed genes. In fact, mRNA levels of several complex subunits (*Mtnd4, Mtnd5*, *Mt-Cyb*, *Mtco1, Mt-Co3)* and rRNA 12S (*Mt-Rnr1*) were significantly higher in senescent cells than in non-senescent cells (Supplementary Fig. S2 online), supporting mitochondrial biogenesis^10,27,28^. *Mfn1* silencing did not affect the expression of these mitochondrial genes but significantly increased the expression of *Mtatp8* and *Mtatp6* (Supplementary Fig. S2 online). Differences with reports from other authors are probably due to the cell type, or the dose and time of exposure to TMZ resulting in cytotoxicity or chemoresistance^23,24^, instead of senescence.

### Protein secretion by melanoma cells

To characterize the secretome of therapy-induced senescent melanoma cells, and understand the impact of *Mfn1* silencing in protein secretion, we performed quantitative shotgun proteomics by LC–MS/MS. We identified 957, 1383, 715 and 1157 proteins in the culture media of shScr DMSO, shScr TMZ, shMfn1 DMSO and shMfn1 TMZ cells, respectively, detected in three replicates per condition (Supplementary Dataset S1 online); analysis with DAVID v6.8 and the Gene Ontology Cellular Component database confirmed that the majority of the proteins (74, 66, 73 and 67%, respectively) could be found in the extracellular compartment (Supplementary Dataset S1 online). Using Enrichr and the “Jensen Compartment” database, we confirmed that the media of the four conditions was significantly enriched in proteins from the terms “Extracellular region” and “Extracellular vesicle”, including “Extracellular exosome” (Supplementary Dataset S1 online).

Differentially expressed proteins were visualized by a heatmap with unsupervised hierarchical clustering (Fig. 1g), in which biological replicates were tightly clustered, and a clear separation between conditions was apparent. Principal component analysis (PCA, Fig. 1h) showed differences between senescent (TMZ-treated) and non-senescent cells (DMSO-treated) in the first dimension. While in the second-dimension, samples were separated depending on the presence of mitofusin 1 (Fig. 1h).

To control for changes in protein composition due to cell death we measured lactate dehydrogenase activity, a cytoplasmic enzyme that is released to the extracellular media when the plasma membrane is damaged. Lactate dehydrogenase activity in the culture media was approximately three- fold higher in cells exposed to TMZ, than in those treated with the vehicle. However, no significant differences were found between cells with and without mitofusin 1 before or after treatment (Supplementary Fig. S1 online). Similar results were obtained assessing cell death and viability by dye exclusion with Trypan blue (Supplementary Fig. S1 online).

Overall, our results suggest that *Mfn1* silencing impacts on the extracellular protein composition of both senescent and non-senescent cells, and the differences observed are probably due to changes in protein secretion and not cell death.

### Mfn1 silencing affects protein secretion by non-senescent melanoma cells

We then analyzed the impact of *Mfn1* silencing on the extracellular proteome of non-senescent melanoma cells. Using the Venn diagram module of the PatternLab software we found the proteins exclusively detected in shScr DMSO or shMfn1 DMSO (P < 0.05). Only 15 proteins were exclusively found in cells without mitofusin 1, while 149 could be identified only in control (shScr DMSO). Regarding the proteins found in both conditions, using the PatternLab TFold module, we determined that 64 were increased in shMfn1 DMSO cells, while 145 were found in higher levels in shScr DMSO cells (Supplementary Fig. S3 and Supplementary Dataset S2A online). These results suggest that *Mfn1* silencing reduces secretome diversity in non-senescent cells.

Interestingly, enrichment analysis with the Reactome data base, revealed that the term “Immune system” (R-HSA-168256) was significantly decreased, presenting adjusted P values (Padj) of 5 × 10^–10^, in shMfn1 DMSO cells with respect to shScr DMSO cells (Supplementary Dataset S2B online). Melanoma cells are reported to modulate the tumor microenvironment, avoiding the immune response^29^, thus *Mfn1* silencing could affect tumor development.

### Secretome composition and pathway analysis in senescent versus non-senescent cells

**Figure 2:**
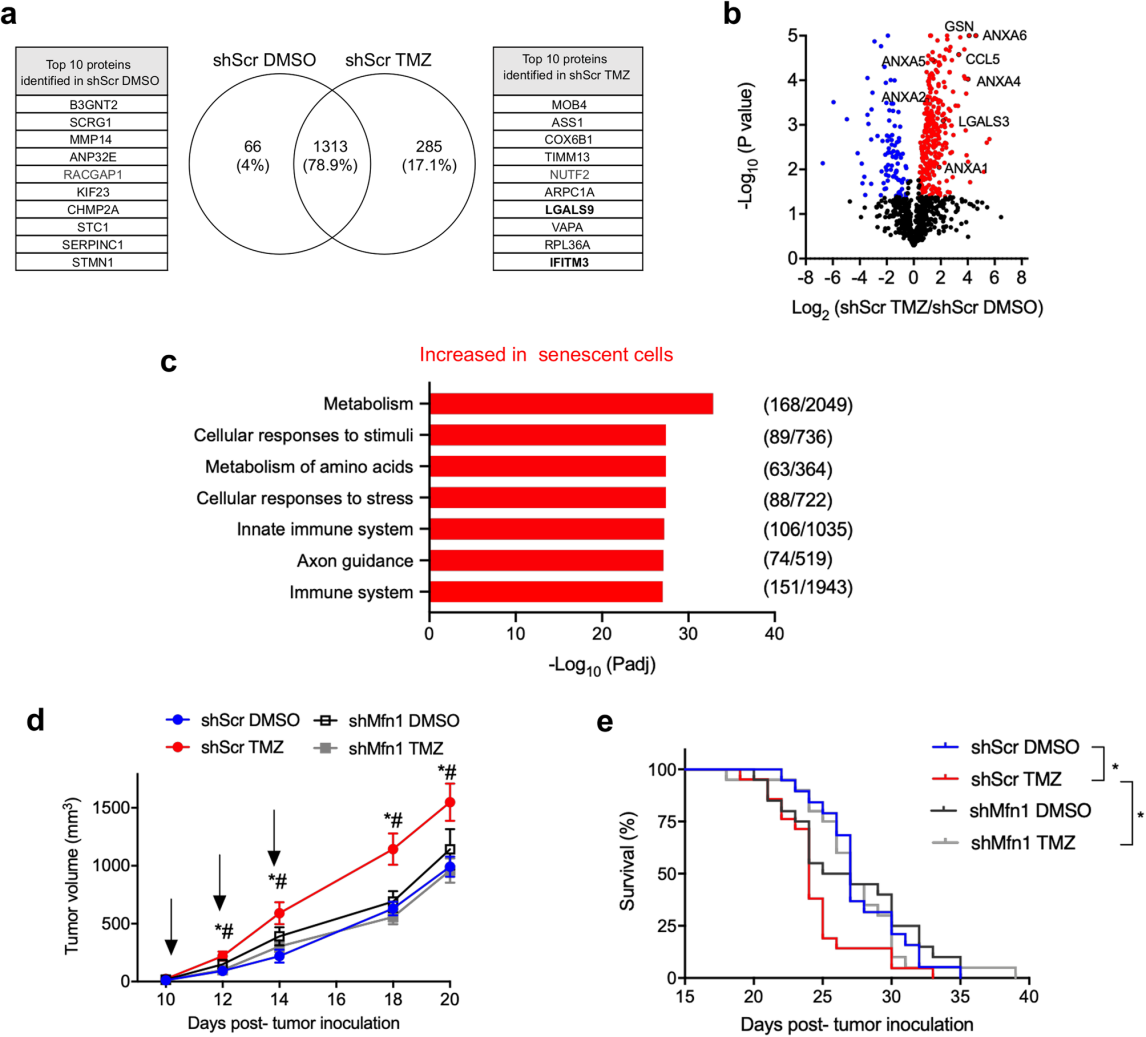
Analysis of the senescence associated secretory phenotype (SASP) of melanoma cells treated with a DNA damaging agent. (**a**) Conditioned media was obtained from shScr cells exposed to TMZ or DMSO, and quantitative analysis of the proteins identified in both conditions using the shotgun approach was performed. The Venn diagram shows proteins statistically exclusively detected in shScr DMSO (non-senescent) or shScr TMZ (senescent) cells (P < 0.05). The tables show the 10 top proteins exclusively found in each condition (complete list in Supplementary Dataset S3A). Selected differential proteins discussed in the text are highlighted in bold. (**b**) Volcano plot showing proteins with differential abundance between conditions. Each dot represents a protein identified in at least four of the six biological replicates, red and blue dots represent proteins significantly increased or decreased, respectively, in shScr TMZ cells vs shScr DMSO cells (BH q-value < 0.05) (the identity of each dot is shown in Supplementary Dataset S3A). Selected differential proteins discussed in the text are labeled. (**c**) Pathway enrichment analysis with the Reactome database, considering the proteins present exclusively in senescent cells (shScr TMZ) or significantly increased in senescent (shScr TMZ) vs non senescent cells (shScr DMSO) (complete list in Supplementary Dataset S3B). The relation between the number of proteins from the sample and the number of proteins found in the term is shown in brackets. (**d**) Mice were injected subcutaneously with 2.5 × 10^5^ cells in the right flank. On days 10, 12 and 14 p.t.i. conditioned media (100 μl) from shScr DMSO, shMfn1 DMSO, shScr TMZ or shMfn1 TMZ was injected intratumorally (arrows), and tumor size was measured. Results show the mean ± SE (n = 23–25, per group). Two-way ANOVA and Tukey post-hoc for multiple comparisons *P < 0.05, with respect to shScr DMSO, ^#^P < 0.05, with respect to shMfn1 TMZ. (**e**) Survival curves for the animals treated as described in (**d**). Log-rank test, curve comparison *P < 0.05, (n = 23–25).

Then we compared the composition of the senescent and non-senescent secretomes from melanoma cells exposed to TMZ or vehicle (shScr TMZ vs shScr DMSO). We identified proteins exclusively detected in the media of senescent cells (285 proteins) and non-senescent cells (66 proteins) (P < 0.05) (Fig. 2a and Supplementary Dataset S3A online). Regarding the proteins present in both conditions, we determined that 306 proteins were significantly increased in senescent with respect to non-senescent cells and 94 were significantly decreased (Fig. 2b and Supplementary Dataset S3A online). Overall, senescent cells presented a more diverse secretome than non-senescent cells, and an increase in extracellular levels of several proteins, supporting the acquisition of a secretory phenotype.

Enrichment analysis with the Reactome database, showed a significant enrichment in several pathways for proteins exclusively detected or increased in senescent versus non-senescent cells (Fig. 2c and Supplementary Dataset S3B). We focused on the “Immune system” pathway (R-HSA-168256) because immunomodulation of tumor microenvironment, for example by immunotherapies, represents one of the main treatments for melanoma. Several proteins present in the “Immune system” pathway (Supplementary Dataset S3B) were reported to affect cancer development, generating an immunosuppressive tumor microenvironment and promoting resistance to therapies, including galectin-9 (LGALS9, Fig. 2a)^30–32^, interferon-induced transmembrane protein 3 (IFITM3, Fig. 2a)^33^, gelsolin (GSN, Fig. 2b)^34^, chemokine (C–C motif) ligand 5 (CCL5, Fig. 2b)^35,36^, galectin-3 (LGALS3, Fig. 2b)^37,38^, and annexins (ANXA) 1, 2, 4, 5 and 6 (Fig. 2b)^39^. Additionally galectin-9 and interferon-induced transmembrane protein 3 (Fig. 2a) were described as potential prognosis biomarkers of an unfavorable outcome in cancer^30,31,33,40^.

### Mfn1 silencing modifies the SASP

Previous work by our group showed that *Mfn1* silencing reduced IL-6 secretion by senescent cells^10,^ therefore we analyzed if a similar effect was observed for other proteins. Comparative analysis of the secretome of shMfn1 melanoma cells treated with TMZ or DMSO was performed as described before (Supplementary Fig. S3 and Supplementary Dataset S4A online). Pathway analysis showed that the term “Immune system” (Reactome R-HSA-168256, Supplementary Dataset S4B) was enriched in shMfn1 TMZ cells with respect to shMfn1 DMSO (Padj = 4 × 10^–30^) as observed for shScr cells with and without exposure to the drug (Fig. 2c).

We then compared the extracellular proteome of senescent cells (shScr TMZ) and senescent cells without mitofusin 1 (shMfn1 TMZ) (Supplementary Fig. S3 and Supplementary Dataset S5A). Pathway enrichment analysis showed that the term “Immune system” (Reactome R-HSA-168256) was significantly lower in shMfn1 TMZ cells with respect to shScr TMZ cells (Padj = 8 × 10^–14^) (Supplementary Dataset S5B).

To identify which proteins secreted by senescent cells were affected by *Mfn1* silencing we looked at pairwise comparisons of shScr TMZ vs shScr DMSO, shMfn1TMZ vs shMfn1 DMSO and shMfn1 TMZ vs shScr TMZ. We noted that the potentially immunosuppressive proteins mentioned above (galectin-9, IFITM3 3, ANXA6, ANXA4, GSN, and CCL5) were exclusively detected or increased in senescent cells (shScr TMZ) versus non-senescent cells (shScr DMSO) and were reduced when mitofusin 1 was lacking (Table 1).

**Table 1:** Silencing *Mfn1* impacts on protein secretion by senescent cells.

ID	Protein (gene)	Normalized XIC (× 10^–5^)												shScr DMSO vs shScr TMZ*	shMfn1 DMSO vs shMfn1 TMZ*	shScr TMZ vs shMfn1 TMZ*
		shScr DMSO			shScr TMZ			shMfn1 DMSO			shMfn1 TMZ					
		1	2	3	1	2	3	1	2	3	1	2	3			
O08573/G3X9T7	Galectin-9 (*Lgals9*)	0	0	0	15	9	12	0	0	0	3	0	3	EX shScr TMZ (P < 0.05)	EX shMfn1 TMZ (P < 0.05)	EN shScr TMZ (P = 9 × 10^–3^)**
Q9CQW9	Interferon-induced transmembrane protein 3 (*Ifitm3*)	0	0	0	11	10	10	0	0	0	0	0	0	EX shScr TMZ (P < 0.05)	-	EX shScr TMZ (P < 0.05)
P14824	Annexin A6 (*Anxa6*)	0.4	0.9	1	16	18	19	0	0	0	6	4	4	EN shScr TMZ (P = 1 × 10^–5^)	EX shMfn1 TMZ (P < 0.05)	EN shScr TMZ (P = 6 × 10^–5^)
P13020	Gelsolin (*Gsn*)	2	4	3	46	44	50	0.3	0	1	31	30	34	EN shScr TMZ (P = 1 × 10^–5^)	EN shMfn1 TMZ (P = 2 × 10^–4^)	EN shScr TMZ (P = 1 × 10^–3^)
P97429/D3Z0S1	Annexin A4 (*Anxa4*)	1	2	1	20	26	22	0	0	0	8	4	4	EN shScr TMZ (P = 9 × 10^–5^)	EX shMfn1 TMZ (P < 0.05)	EN shScr TMZ (P = 5 × 10^–4^)
P30882	C–C motif chemokine 5 (*Ccl5*)	20	10	7	124	134	118	5	7	5	82	82	78	EN shScr TMZ (P = 3 × 10^–5^)	EN shMfn1 TMZ (P = 1 × 10^–5^)	EN shScr TMZ (P = 4 × 10^–4^)

Selected proteins increased or exclusive detected in senescent cells (shScr TMZ), with respect to non-senescent cells (shScr DMSO), that were affected by *Mfn1* silencing. The table shows normalized xic values for each sample, and the existence of significant differences between conditions.

*EX* exclusively detected, *EN* enriched.

*P values were calculated by Patternlab’s Venn or T-fold modules.

**This identification was filtered out by Patternlab for Proteomics T-fold analyzer due to its low abundance.

Overall, these results suggest that *Mfn1* silencing reduced the secretion of proteins that could affect the immune response towards the tumor. We reasoned that immunomodulatory proteins secreted by senescent cells would affect tumor progression, and that *Mfn1* silencing might mitigate these effects. To test this hypothesis tumors were generated in C57BL/6 mice by subcutaneous inoculation of B16-F1 melanoma cells, a well-established animal model of human melanoma^41^. At day 10 post-tumor inoculation (p.t.i.), when tumors became palpable, we injected conditioned media from cells in different conditions (non-senescent and senescent cells with or without out *Mfn1* silencing) (depicted as arrows in Fig. 2d). Treatment with conditioned medium from senescent cells (shScr TMZ) resulted in a significant increase in tumor size and decreased survival, with respect to animals treated with medium from control cells (shScr DMSO) (Fig. 2d,e). This pro-tumoral effect was not observed when tumors were treated with conditioned medium from senescent cells lacking mitofusin 1 (shMfn1 TMZ) (Fig. 2d,e).

### Mfn1 silencing reduces Galectin-9 secretion by senescent cells

**Figure 3:**
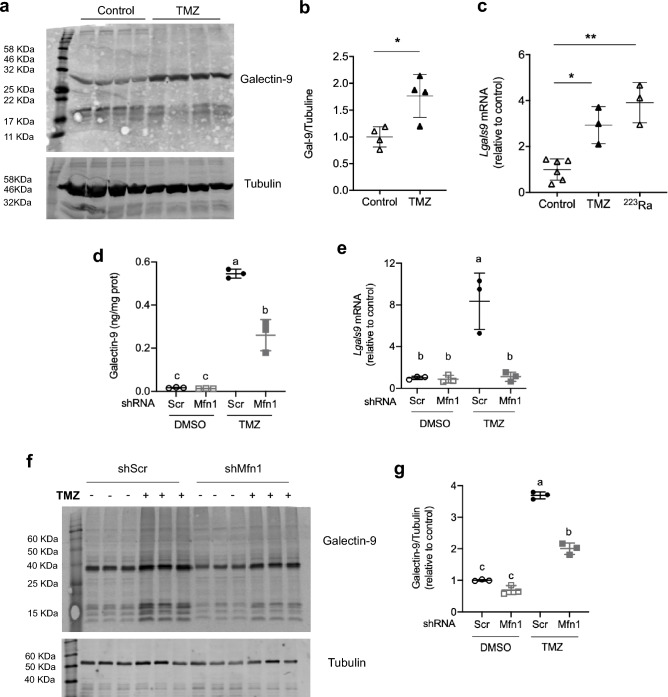
*Mfn1* silencing reduces galectin-9 expression and secretion in senescent cells. (**a**–**c**) B16-F1 cells were treated with DMSO, TMZ or ^223^Ra: (**a**) representative Western-Blot (WB) of intracellular levels of galectin-9. (**b**) Quantification of the WB in (**a**), galectin-9 was normalized using tubulin as loading control and results were expressed relative to control condition. Unpaired t-test, two tails, *P = 0.014 (n = 4). (**c**) *Lgals9* mRNA reported relative to control value. One-way ANOVA (P = 0.0003), Tukey post-hoc for multiple comparisons, *P = 0.005, **P = 0.0003 vs control (n = 3–6). (**d**–**g**) ShScr and shMfn1 cells were treated with TMZ or DMSO: (**d**) Galectin-9 secretion assessed by ELISA. Two-way ANOVA, main effects of treatment (P < 0.0001), shRNA (P = 0.0002) and their interaction (P = 0.0002). Tukey post-hoc for multiple comparisons, different letters are significantly different (P ≤ 0.0002). (**e**) *Lgals9* mRNA, reported relative to control value. Two-way ANOVA, main effects of treatment (P = 0.0015), shRNA (P = 0.002) and their interaction (P = 0.002). Tukey post-hoc for multiple comparisons, different letters are significantly different (P ≤ 0.0009). (**f**) Representative western-blot of intracellular levels of galectin-9. (**g**) Quantification of protein levels in (**f**). Galectin-9 was normalized using tubulin as loading control and expressed relative to control condition. Two-way ANOVA, main effects of treatment (P < 0.0001), shRNA (P < 0.0001) and their interaction (P < 0.0001). Tukey post-hoc for multiple comparisons, different letters are significantly different (P < 0.0001). Results are the mean ± SD (n = 3–5). The uncropped images of the blots, with different degrees of exposure, can be found in Supplementary Figs. S10 and S11.

We decided to validate the increase in galectin-9, since it is elevated in plasma of patients with metastatic melanoma and correlates with poor survival^30^. We confirmed the increase of galectin-9 protein and *Lgals9* mRNA levels in B16-F1 cells exposed to TMZ (Fig. 3a–c). To expand this observation we incubated the cells with the alpha-particle emitter ^223^Ra, another efficient DNA damaging agent used for treating bone metastases^42,43^. Incubation with ^223^Ra increased the percentage of SA-β-Gal positive cells with respect to the control (56 ± 17% versus 11 ± 8%, respectively, t-test P = 0.003, n = 4), as well as the expression of *Cdkn1a* (p21) (11 ± 1 versus 1 ± 0.1, t-test P = 0.01, n = 4) and *Ccl5,* a component of the SASP (93 ± 34 versus 1 ± 0.2, t-test P = 0.001, n = 4), indicating induction of senescence in the culture. The expression of *Lgals9* was also increased in senescent cells induced by exposure to this radionucleoid (Fig. 3c).

Galectin-9 could not be detected in the media of non-senescent cells, shScr DMSO or shMfn1 DMSO, by proteomic techniques (Table 1). Though its levels increased upon exposure to TMZ in both cell lines the abundance was significantly lower in the media of cells lacking mitofusin 1 (Table 1).

We validated these results measuring galectin-9 in the culture media by ELISA, confirming that it was only detected in senescent cells, and that the lack of mitofusin 1 significantly reduced its concentration (Fig. 3d). However, at the intracellular level the protein could be found in both senescent and non- senescent cells, and its levels rose upon exposure to TMZ, but were significantly lower when *Mfn1* was silenced (Fig. 3f,g). Cleaved forms of galectin-9 of lower molecular weight, could be observed in the Western-blots with a similar pattern as that reported for galectin-9 cleavage by proteases metalloproteinase-3 and elastase^44^ (Fig. 3a,f). Similar results were obtained for *Lgals9* expression, that was increased in senescent versus non-senescent cells, and negatively affected by *Mfn1* silencing (Fig. 3e). Interestingly, the intracellular protein and mRNA levels of this lectin were similar in non-senescent cells (shScr DMSO and ShMfn1 DMSO) suggesting that *Mfn1* is only required for the increase in expression, protein synthesis and secretion in senescent cells. These results were confirmed using a second shRNA (shMfn1_#2) to silence *Mfn1* (Supplementary Fig. S4 online).

We also assessed if *Mfn1* silencing affected the expression of other cytokines and chemokines secreted by senescent melanoma cells^10,45^. Treatment with TMZ increased mRNA levels of *Ccl5, Il6, Ccl2*, and *Tgfb,* but *Mfn1* silencing did not reduce their mRNA levels significantly (Supplementary Fig. S5). These results were surprising, since lack of mitofusin 1 reduced the secretion of both IL-6^10^ and CCL5 (Table 1) and suggested regulation at a posttranslational level in opposition to our observations for galectin-9.

### Mfn1 silencing delays tumor development.

**Figure 4:**
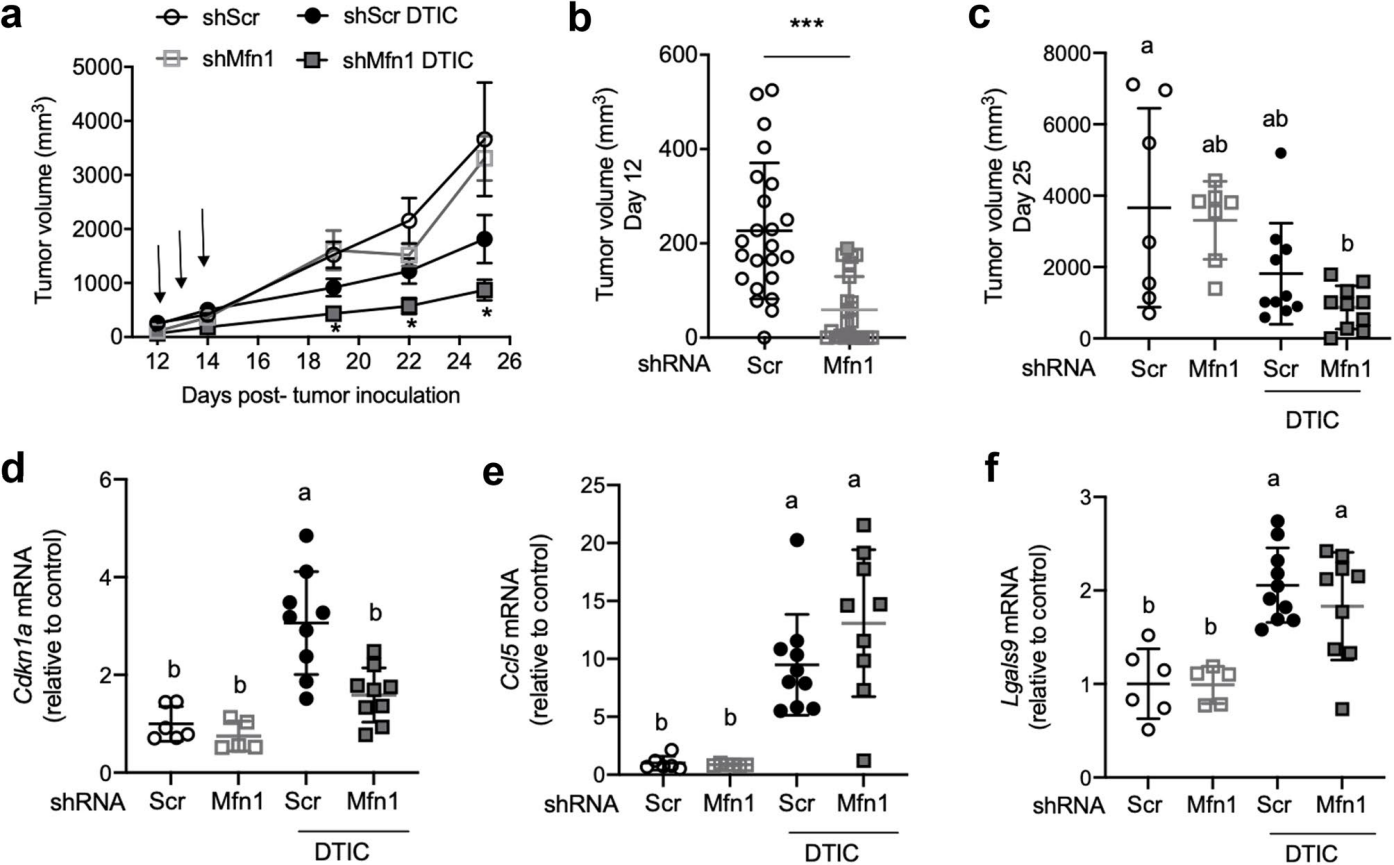
Mfn1 silencing improves the response to chemotherapy with a DNA damaging agent in a mice model of melanoma. shScr or shMfn1 B16-F1 cells were injected subcutaneously in the right hind leg of the mice, and when tumors became palpable mice were treated with three doses of DTIC (arrows). (**a**) Tumor size was measured at different time points p.t.i.. Two-way ANOVA with Tukey post-hoc analysis with respect to control (shScr) * P ≤ 0.01. Results are the mean ± SEM (n = 10). (**b**) Day 12 p.t.i., tumor volume before DTIC treatment. Unpaired t-test, two tails ***P < 0.0001 (n = 23). (**c**) Day 25 p.t.i., tumor volume after treatment. Two-way ANOVA, main effects of treatment (P = 0.0005), shRNA (P = 0.3) and their interaction (P = 0.6). Tukey post-hoc for multiple comparisons, different letters are significantly different (P ≤ 0.02). (**d**–**f**) mRNA levels of senescence marker or SASP components in the tumor. Two-way ANOVA (main effects of treatment, shRNA and their interaction) and Tukey post-hoc for multiple comparisons, different letters are significantly different: (**d**) *Cdkn1a* (p21) in the tumors (P < 0.0001, P = 0.004, P = 0.03), Tukey post-hoc P ≤ 0.0008. (**e**) *Ccl5* (P < 0.0001, P = 0.3, P = 0.2), Tukey post-hoc P ≤ 0.0036. (**f**) *Lgals9* (P < 0.0001, P = 0.5, P = 0.5), Tukey post-hoc P ≤ 0.009. (**b**–**f**) Results are the mean ± SD (n = 10).

We then explored if *Mfn1* silencing could affect tumor development (Fig. 4a). We generated tumors with melanoma cells with and without mitofusin 1, by injecting shScr or shMfn1 cells subcutaneously in the hind leg of the mice. We observed that *Mfn1* silencing resulted in a smaller tumor volume even in the absence of treatment (day 12 p.t.i., Fig. 4b). No differences in cell growth or migration, assessed by the wound healing assay, were found between shScr and shMfn1 cell lines in culture conditions in vitro that could explain the smaller size of shMfn1 tumors (Fig. 1e and Supplementary Fig. S6 online).

To determine the impact of *Mfn1* silencing on the response to chemotherapy, half of the animals in each group were treated with dacarbazine (DTIC), a TMZ analogue that produces the same methylating agent (3-methyl-(triazen-1-yl)imidazole-4-carboxamide) after activation in the liver^13,46,47^. When tumors became palpable (day 12 p.t.i.) mice were administered intraperitoneally three consecutive doses of DTIC^48^, and tumor size was measured. Remarkably, we only observed a significant decrease in tumor size, with respect to the control group (shScr), in the shMfn1 DTIC group (Fig. 4a,c). In spite of the delay observed in tumor growth, survival curves did not show a significant impact of *Mfn1* silencing in overall survival. The treatment with DTIC increased survival, but this was not improved by *Mfn1* silencing (Supplementary Fig. S7 online).

To verify that DTIC treatment could induce senescence in vivo, the tumors were excised at day 25 p.t.i. and we looked for markers of senescence. We observed a significant increase in *Cdkn1a* (p21) expression in shScr TMZ vs shScr DMSO that was not present when *Mfn1* was silenced (Fig. 4d). The expression of *Ccl5* and *Lgals9* was also increased in the tumors of DTIC-treated animals (shScr DTIC and shMfn1 DTIC) with respect to the control condition (shScr) (Fig. 4e,f). However, we did not detect differences in the mRNA levels of these factors between tumors with and without mitofusin 1 (Fig. 4e,f), maybe because other immune cells present in the tumor can also express *Ccl5* and *Lgals9.*

**Figure 5:**
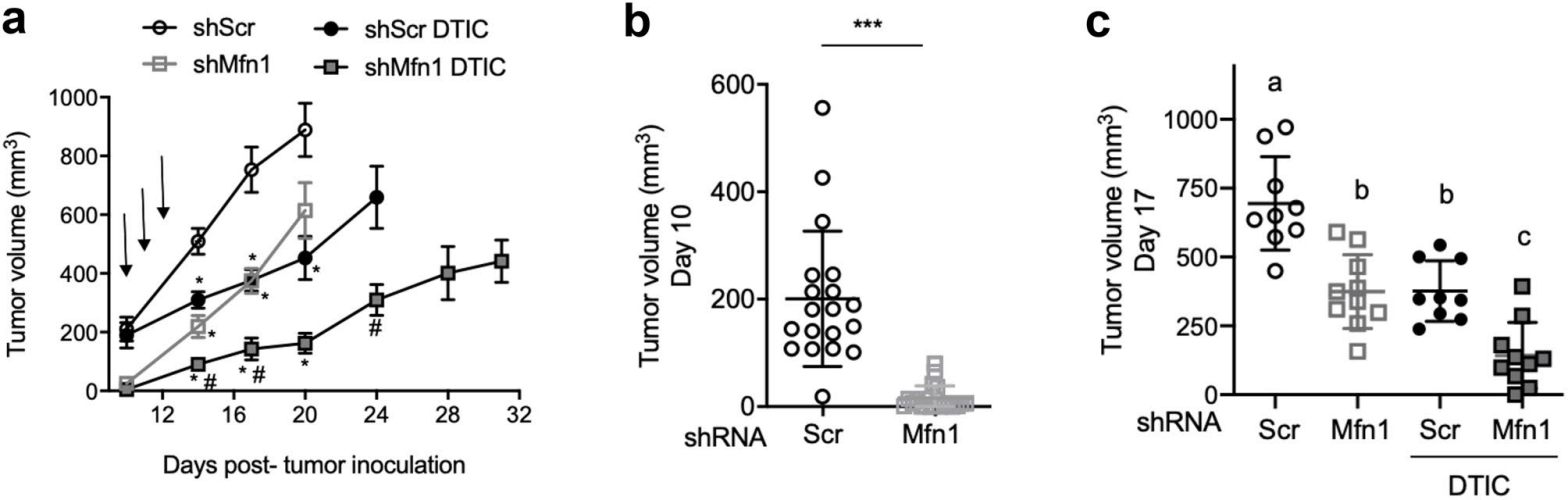
*Mfn1* silencing delays tumor growth. shScr or shMfn1 B16-F1 cells were injected intradermically in the right hind leg of the mice, when tumors became palpable mice were treated with three doses of DTIC. (**a**) Tumor size was measured every 2–3 days and differences in tumor size were evaluated at different time points p.t.i.. Results show the mean ± SEM (n = 10, per group). Two-way ANOVA with Tukey post-hoc analysis, with respect to control shScr *P ≤ 0.003 or shScr DTIC #P ≤ 0.006. (**b**) Day 10 p.t.i., prior to DTIC treatment. Unpaired t-test, two-tails ***P < 0.0001 (n = 20, per group). (**c**) Day 17 p.t.i.. Two-way ANOVA, main effects of treatment (P < 0.0001), shRNA (P < 0.0001), and their interaction (P = 0.2). Tukey post-hoc for multiple comparisons, different letters are significantly different (P ≤ 0.02). (**b**,**c**) Results show the mean ± SD (n = 10, per group).

We also analyzed what happened with tumors implanted at the intradermal level. Again, we noticed that lack of mitofusin 1 resulted in smaller tumors (Fig. 5a,b). Mfn1 silencing per se (shMfn1) delayed tumor growth until day 17 p.t.i. (Fig. 5a,c). Similarly, DTIC treatment alone (shScr DTIC) resulted in smaller tumors than the control (shScr) until day 20 p.t.i. (Fig. 5a,c). Interestingly, *Mfn1* silencing further delayed tumor development in treated animals, and tumor size was significantly smaller in shMfn1 DTIC mice than in all the other conditions (Fig. 5a,c). In this model, *Mfn1* silencing increased the survival of DTIC treated animals (Supplementary Fig. S7 online).

### *Mfn1* silencing increases tumor immune cell recruitment

**Figure 6:**
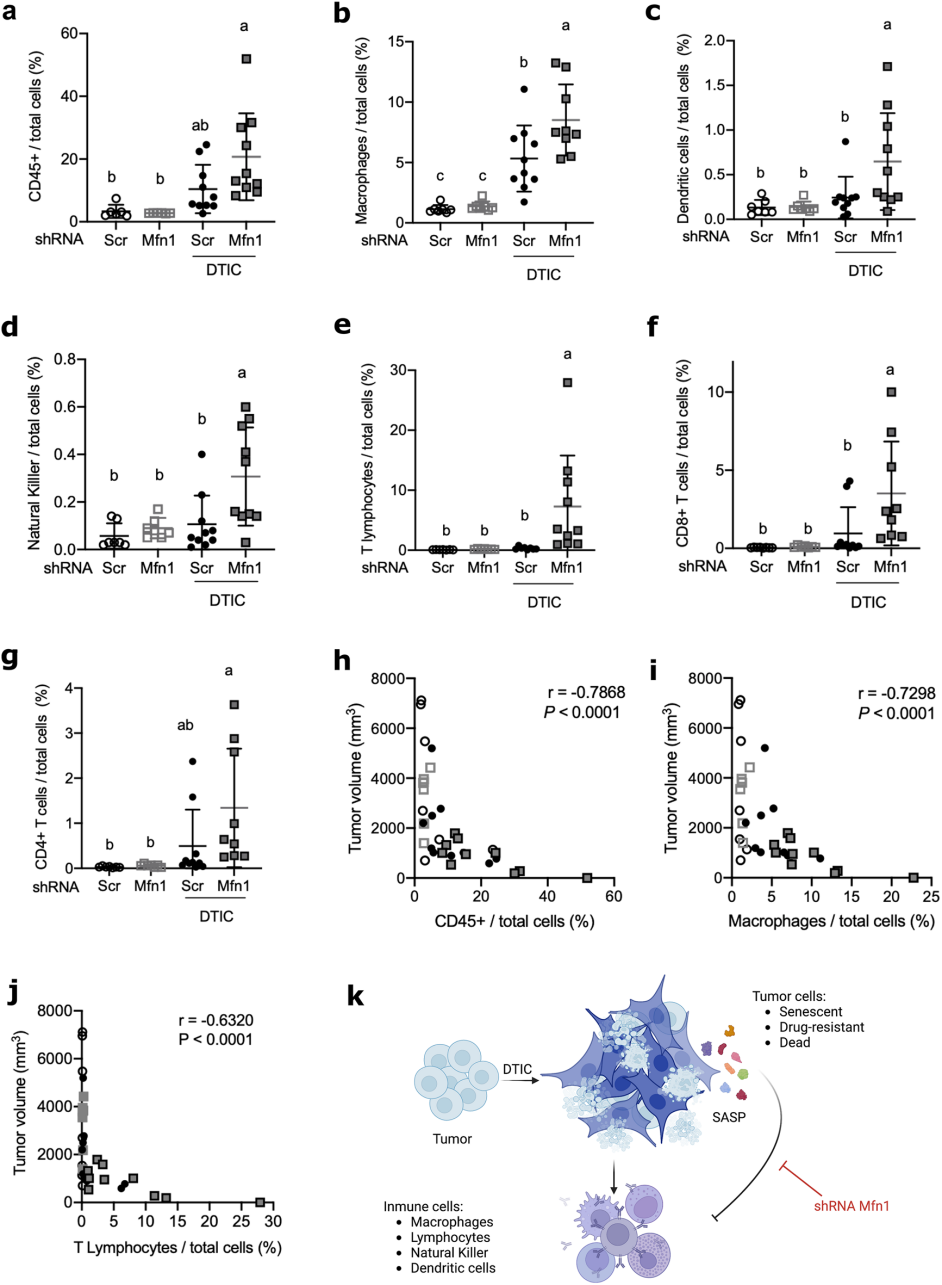
*Mfn1* silencing increases immune cell recruitment to the tumor in mice undergoing chemotherapy. ShScr or shMfn1 cells were injected subcutaneously in the right hind leg of the mice, when tumors became palpable, on day 12 p.t.i., mice were treated with three doses of DTIC. On day 25 p.t.i. tumors were excised and analyzed by flow cytometry. Gating strategies for flow cytometry analysis are shown in Supplementary Figs. S4 and S5 online. (**a**–**g**) Percentage of the different immune cell populations infiltrating the tumor. Results show the mean ± SD (n = 8–12, per group). Statistical tests: two-way ANOVA (main effects of treatment, shRNA and their interaction) and Tukey post-hoc for multiple comparisons, different letters are significantly different: (**a**) Immune cells (CD45 +) (P = 0.0007, P = 0.15, P = 0.11), Tukey post-hoc (P < 0.005). (**b**) Macrophages (P < 0.0001, P = 0.03, P = 0.06), Tukey post-hoc P ≤ 0.02. (**c**) Dendritic cells (P = 0.01, P = 0.08, P = 0.09). Tukey post-hoc P < 0.05. (**d**) NK cells (P = 0.008, P = 0.02, P = 0.08), Tukey post-hoc P ≤ 0.01. (**e**) T lymphocytes (P = 0.04, P < 0.05, P = 0.05), Tukey post-hoc P = 0.03. (**f**) CD8 + T cells (P = 0.003, P = 0.06, P = 0.07), Tukey post-hoc P ≤ 0.04. (**g**) CD4 + T cells (P = 0.003, P = 0.1, P = 0.1), Tukey post-hoc P ≤ 0.01. (**h**–**j**) Correlation analysis for tumor volume (from Fig. 4c) and immune cell infiltration. Spearman correlation coefficient (r) and P are shown in the figures: (**h**) tumor volume and CD45 + immune cells. (**i**) Tumor volume and macrophage infiltration. (**j**) Tumor volume and T cell infiltration. (**k**) Schematic representation of tumor cells in animals treated with DTIC, resulting in cell death, senescence and drug resistant surviving cells. Dead cells release molecules that can attract immune cells, but senescent cells can secrete immunomodulatory molecules. *Mfn1* silencing with shRNA reduces the SASP favoring the recruitment of immune cells to the tumor. This figure was created with BioRender.com.

Galectin-9 and several of the identified SASP components had been reported to play immunosuppressive roles in the tumor microenvironment^30–32,49^. Thus, we explored if *Mfn1* silencing would affect immune cell recruitment to the tumor. Mice were inoculated subcutaneously with melanoma cells (shScr or shMfn1) and treated with DTIC as described above. On day 25 p.t.i. the tumors were excised and the different populations of immune cells infiltrating the tumors were determined by flow cytometry (Fig. 6 and Supplementary Figs. S8 and S9 online).

B16-F1 cells are not very immunogenic^50^ and only a low percentage of CD45 + immune cells could be detected in the tumors of untreated animals (Fig. 6a). Treatment with DTIC resulted in an increase in the percentage of immune cells in the tumor. However, significant differences with respect to the control condition (shScr) could only be found in animals carrying tumors lacking mitofusin 1 and treated with DTIC (shMfn1 DTIC) (Fig. 6a). It is worth noting that the opposite profile was observed when analyzing tumor size, since only tumors from the shMfn1 TMZ group were significantly smaller than those of the control (shScr) group (Fig. 4c). In fact, a strong negative correlation was observed between tumor volume and CD45 + cell infiltration (Fig. 6h).

Analysis of cells of the myeloid lineage revealed an increase in macrophage infiltration, in both shScr and shMfn1 tumors after treatment with DTIC, with a significantly higher percentage in those lacking mitofusin 1 (ShMfn1 DTIC) (Fig. 6b). Dendritic cells were significantly higher only in tumors lacking mitofusin 1 after treatment (ShMfn1 DTIC) with respect to the control condition (shScr) (Fig. 6c). Negative correlations could be observed between tumor size and the percentage of macrophages (Fig. 6i); as well as for tumor size and the percentage of dendritic cells infiltrating the tumor (Spearman correlation coefficient r = − 0.6958, P < 0.0001). Regarding macrophage polarization, we observed a significant increase in classical M1/alternative M2 ratio in senescent cells without mitofusin 1 with respect to senescent cells with mitofusin 1 (3 ± 1 versus 1.5 ± 0.8, P = 0.04, n = 10 per group).

Similar results were obtained for cells from the lymphoid lineage. Natural killer (NK) cells, CD4 + and CD8 + T lymphocytes were significantly increased with respect to the control (shScr), only in the tumors were *Mfn1* was silenced and the animals were treated with DTIC (shMfn1 DTIC) (Fig. 6d–g). Negative correlations could be observed between tumor size and the percentage of Natural killer cells (Spearman correlation coefficient r = − 0.6240, P < 0.0001), as well as for tumor size and T cell infiltration (Fig. 6j).

Overall, our results suggest that chemotherapy with DTIC can induce senescence as well as tumor cell death. Dying cancer cells will produce molecules that attract and activate immune cells. However, senescent cells can produce immunomodulatory proteins, such as galectin-9, that will hamper the recruitment of immune cells to the tumor site. We propose that *Mfn1* silencing can reduce the secretion of these immunosuppressive factors, increasing the percentage of immune cells in the tumor and delaying tumor development (Fig. 6k).

## Discussion

Senescent cells can be formed during tumorigenesis or after cancer treatment, and both pro- and anti-tumoral roles have been assigned to the SASP is this context^9,51^. In melanoma DNA damaging chemotherapeutics induce cell death^46^ but can also induce senescence and a SASP, composed of multiple cytokines, metalloproteases and other factors that can impact the tumor microenvironment^10,45,52,53^.

Our proteomic studies also showed that the secretome of melanoma cells exposed to temozolomide contained several immunosuppressive components, capable of enabling tumor development. We also observed that *Mfn1* silencing strongly reduced protein secretion by therapy-induced senescent melanoma cells, including the secretion of several immunomodulatory proteins, identifying a previously unappreciated role for mitofusin 1.

One of the proteins secreted by senescent cells was galectin-9. Increased expression of this lectin was identified by RNA-seq in senescent astrocytes^54^, while a decrease was observed in senescent fibroblasts induced by different stimuli^2,54,55^. To our knowledge galectin-9 secretion by senescent cell has not been reported. Galectin-9 gene expression increased upon cell exposure to DNA-damaging agents TMZ and ^223^Ra at doses similar to those found in patients under treatment^56–58^. Galectin-9 is secreted by cancer and myeloid cells upon induction by interferon β and γ (IFNβ and IFNγ) and has immunosuppressive roles in the tumor microenvironment^31^. It is reported to reduce natural killer (NK) cytotoxicity and cytokine production^49^, promote apoptosis of CD4 + and CD8 + T cells, and CD14 + monocytes^30,31^, increase regulatory T cells (Treg)^30,59^, T helper 2 cells (Th2) and M2 macrophages^30,60^, impairing the antitumor immune response. On the other hand, galectin- 9 has also been reported to suppress metastasis in melanoma and colon carcinoma mice models^61^. Galectin-9 expression and secretion were downregulated by *Mfn1* silencing.

Other immunomodulatory proteins from the senescent secretome affected by *Mfn1* silencing were gelsolin, reported to promote apoptosis of antitumor M1 macrophages and CD8 + T cells, and CD4 + polarization to Th cells^34^; chemokine CCL5 known to induce CD8+ T cell apoptosis and recruitment of Treg cells^35,36^; galectin-3 capable of promoting T cell apoptosis and tumor resistance to radio and chemotherapy^37,38^; annexins 4 and 6 that can induce a tolerogenic phenotype in dendritic cells^39^; and interferon-induced transmembrane protein 3 an immunomodulatory factor^33^ that can induce paracrine senescence in neighboring cells, further amplifying the SASP^62^. In agreement, injection of conditioned media from senescent cells (shScr TMZ) to the tumors promoted tumor growth, while no effect was observed upon injection of conditioned media from senescent cells lacking mitofusin 1.

Mitofusin depletion was also protective in a different setting when tumors were generated with melanoma cells with or without *Mfn1* silencing and senescence was induced in vivo by treatment with the methylating agent DTIC. Tumors without mitofusin 1 grew more slowly than their control counterparts early after inoculation. In animals subjected to chemotherapy with DTIC, *Mfn1* silencing also affected tumor growth. In particular, when tumors were implanted intradermically, animals of the shMfn1 DTIC group had smaller tumors and higher survival than those of the shScr DTIC group. Our results strongly suggest that this delay in tumor growth could be due to an increase in immune cell recruitment to the tumor. Chemotherapy by itself only produced a significant increase in the percentage of macrophages in the tumor, but when the treatment was accompanied by *Mfn1* silencing an overall increase in recruitment of immune cells (macrophages, DC, NK cells, and T lymphocytes) was observed. Moreover, a significant negative correlation between tumor size and several populations of immune cells was found, being particularly strong for macrophage infiltration. Besides, *Mfn1* silencing per se did not affect melanoma cell growth or migration in culture but had a significant effect in vivo reducing tumor volume. The differences in tumor volume between animals bearing tumors with and without mitofusin 1 were more prominent in intradermal than subcutaneous tumors, maybe because in this site of implantation tumors are more susceptible to the immune response^63^.

Previous studies from our lab showed that TMZ increased the expression of several chemotactic cytokines capable of increasing the recruitment of immune cells, including macrophage chemoattractant cytokines Ccl2, Ccl5 and IL-6^10,35,64^. These cytokines are secreted by senescent melanoma cells and could recruit immune cells to the tumor in DTIC-treated animals. Lack of mitofusin 1 diminished the secretion of IL-6 and Ccl5 by senescent cells and did not affect Ccl2 expression, therefore these cytokines cannot account for the increase in immune cells in ShMfn1 DTIC tumors. On the other hand, immune cell recruitment could be influenced by some of the immunosuppressive proteins secreted by senescent cells that are reduced by *Mfn1* silencing (i.e. IFITM3 and galectin-9). IFITM3-deficient mice secrete inflammatory cytokines that promote immune cell infiltration^33^. While, galectin-9 could impact on immune cell numbers on the tumor by inducing apoptosis of T cells^30,31^. Ccl5, IL-6 and galectin-9 also promote polarization of macrophages from the M1 proinflammatory phenotype to the M2 immunosuppressive protumoral phenotype^30,35,65,66^. These factors are components of the SASP, and *Mfn1* silencing decreases their secretion. In agreement the M1/M2 ratio was higher in tumors of DTIC-treated mice without mitofusin 1.

Melanoma is an aggressive skin cancer of increasing incidence^29^, that is able to suppress or evade the immune system disseminating and forming metastasis^67^. In the metastatic phase, the disease presents a poor prognosis. Targeted therapies and immunotherapies are used as adjuvants and treatment of metastatic melanoma, but resistance to apoptosis and immune system evasion remain a problem. Adjuvant chemotherapies with DNA damaging agents, such as DTIC or TMZ, present a modest response rate and scarce improvement of overall survival in comparison with targeted therapies and immune therapies^29^. Nevertheless, chemotherapies are still used in the palliative treatment of refractory, progressive and relapsed melanoma when immune therapies have failed and targeted therapies cannot be applied^47^.

Chemotherapy-induced senescence is reported to promote both pro- and anti-tumor immunity depending on the setting^9^. Cyclin dependent kinase 4/6 (CDK4/6) inhibitors, in combination with MAPK inhibitors, induced senescence in lung and pancreatic cancer mouse models, and the SASP contributed to a potent antitumor immune response^68,69^. Chemotherapy with cyclophosphamide, also induced senescence and a SASP with anti-tumoral activity in a murine lymphoma model^8,70^. On the other hand, several reports argue that senescent cells generated after chemotherapy can promote tumor development. For example, exposure of fibroblasts to DNA-damaging chemotherapeutics induced senescence and increased secretion of proteins WNT16, SPINK1, and amphiregulin (AREG). These factors affected the prostatic cancer cell phenotype, inducing an epithelial-to-mesenchymal transition (EMT), cancer cell proliferation, invasion and resistance to chemotherapy, as well as increasing PD-L1 expression promoting tumor immune evasion^71–73^. Targeting the SASP components AREG or SPINK1 with specific antibodies enhanced the response to chemotherapy in tumor mouse models^71,73^. Similar results were obtained by Toso et al. that showed that Pten-null senescent cells in prostatic tumors present an immunosuppressive SASP, and treatment with Jak2/STAT inhibitors modified the secretome activating anti-tumor immunity and enhancing the response to chemotherapy^74^. Likewise, selective clearance of senescent cells induced by doxorubicin reduced metastasis and delayed relapse in a breast cancer mouse model^75^. Therefore, developing therapeutical strategies that target senescent cells or the SASP might help enhance the efficiency of these treatments and reduce their toxic effects^75–77^.

Additionally, mitochondria per se appear to be relevant modulators of melanoma immune evasion in non-senescent cells. Gene expression analysis by Laurin et al. showed that PGC-1, master regulator of mitochondrial biogenesis, correlates negatively with the expression of several immunosuppressive proteins, including galectin-9, PD-L1 and PD-L2^78^ in melanoma. Silencing PGC1-beta expression increased galectin-9 levels, and CCL5 secretion in human melanoma cells^78^, suggesting that the mitochondrial biogenesis pathway in melanoma can also modulate the immunosuppressive cancer cell phenotype. However, we must note that senescent melanoma cells did not follow this trend, since our results showed an increase in both mitochondrial biogenesis (including PGC-1 expression)^10^ and secretion of immunosuppressive factors.

Here we present a new strategy to modulate the SASP in therapy-induced senescence. We show that mitofusin 1 silencing reduces the secretion of immunosuppressive components of the SASP, such as galectin-9, generated during chemotherapy avoiding immune system evasion, and increasing chemotherapy efficiency. Mitofusin 2 presents high homology with mitofusin 1 and is also involved in mitochondrial fusion^14^. Previous results showed that *Mfn2* silencing reduced IL-6 secretion by senescent cells^10^, thus these two isoforms appear to play roles in the SASP, but one cannot compensate for the loss of the other. Nevertheless, it is still to be determined if mitofusin 2 is required for the secretion of galectin-9 and other immunosuppressive proteins as observed for mitofusin 1. The role of mitofusins in secretion is not yet understood at the molecular level, but might be related to protein trafficking, organelle tethering, signaling or shifts in nutrient utilization^17,20,21,79^. Silencing mitofusins may have other anti-tumoral consequences, since these proteins also participate in tumor invasion. In cutaneous melanoma, high mitofusin 2 levels are associated with the degree of skin penetration and significantly correlate with lymph node involvement and metastasis^80^. In tumor cells treated with PI3K inhibitor (PX-866), mitofusin 1 participates in mitochondrial migration to the cortical cytoskeleton and is required for cell invasion^81^. Protein secretion is an energy consuming process that also requires correct positioning of mitochondria at plasma membrane sites were exocytosis or vesicle release occur, and near organelles where different stages of the secretion process take place^82,83^.

The development of senotherapeutics, capable of selectively inducing senescent cell death (senolytics) or modulating the SASP (senomorphics), is an area in fast development and its role in cancer treatment is increasingly acknowledged^84,85^. Our results suggest that mitochondrial dynamic proteins are required for secretion and might constitute a new target to improve cancer treatment by DNA damaging agents capable of inducing senescence, reprogramming the SASP, reducing immune system evasion and increasing the response to therapy.

## Methods

### Cell culture

The mouse melanoma B16-F1 cell line (CRL-6323, ATCC) was cultured in Dulbecco’s modified Eagle’s medium (DMEM, Gibco) with 4.5 g/l glucose, 10% FBS (Gibco), penicillin 50 U/ml and streptomycin 50 µg/ml. All cells were maintained at 37 °C in a CO_2_ incubator (95% air, 5% CO_2_). All reagents were from Sigma unless otherwise specified.

### Lentiviral shRNA transduction

Plasmids carrying shRNA constructs targeting *Mfn1,* shMfn1 (TRCN0000081402) and shMfn1_#2 (TRCN0000081398) were from Dharmacon. The plasmid carrying scrambled shRNA was a gift from David Sabatini (Addgene plasmid #1864^86^); pCMV-VSV-G and pCMV-dR8.2 dvpr were a gift from Bob Weinberg (Addgene plasmids #8454 and #8455^87^). Lentiviral vectors were produced in 293 T cells according to protocols established by the Broad Institute RNAi Consortium (www.broadinstitute.org/genome_bio/trc/publicProtocols.html). B16-F1 cells were transduced with lentiviral particles overnight in complete media with 8 μg/ml polybrene. Infected cells were subsequently selected in DMEM supplemented with 2 μg/ml puromycin. In this way we generated cell lines were Mfn1 expression was silenced (shMfn1 and shMfn1_#2), and control cells (shScr).

### ***Treatment with TMZ or ***^***223***^***Ra***

Cell lines were treated with 200 µM TMZ (Sigma) for 5 h, this procedure was performed twice with a 24-h interval, unless otherwise specified. Treatment with the vehicle, dimethyl sulfoxide (DMSO), was used as control in all experiments. Measurements were made five days after the second exposure to the drug, unless otherwise specified.

Alternatively, B16-F1 cells underwent incubation with ^223^Ra at 18 kBq for a period of 48 h, and measurements were made four days after. Under this experimental setup, we performed calculations of mean cellular microdosimetry, operating under the assumption of cell and nuclear diameters measuring 10 and 6 µm, respectively. We employed the calculation model suggested by Roeske and Stinchcomb for determining the absorbed dose of alpha particles, which resulted in 1.3 Gray units (Gy)^88,89^.

### Proteomic analysis by LC–MS/MS

Cultured cells were treated with TMZ as described above. Three biological replicates were prepared per condition. Two days after the last treatment the cells were divided and seeded in 10 cm diameter culture dishes. The next day the cells were washed 3 times with PBS and we added 10 ml of DMEM without FBS. Two days after, conditioned medium was collected and centrifuged at 10,000*g* to remove cell debris. Protein concentration was determined by the bicinchoninic acid assay.

Proteins were run on 1 cm long SDS gels (12% acrylamide), in-gel cysteine alkylation was performed by incubation with 10 mM dithiothreitol for 1 h at 56 °C followed by incubation with 55 mM iodoacetamide at room temperature for 45 min. In-gel digestion of 1 cm protein bands was performed overnight at 37 °C by incubation with trypsin (Promega) 1:50 enzyme:protein (w:w) in ammonium bicarbonate 50 mM. Peptide extraction was performed with 0.1% trifluoroacetic acid (TFA) in 60% acetonitrile as previously described^90^. Samples were vacuum-dried, resuspended in 0.1% formic acid, and desalted using Ziptip® C18 (Millipore).

Tryptic peptides were separated using a nano-HPLC (UltiMate 300, Thermo Scientific) coupled to hybrid mass spectrometer Q-Orbitrap (QExactive Plus, Thermo Scientific). Each biological replicate (3 μg) was injected twice into an Acclaim PepMap TM 100 C18 pre column (75 μm × 2 cm, 3 μm, Thermo Scientific) and separated on a PepMap TM RSLC C18 analytical column (75 μm × 50 cm, 2 μm, 100 Å, Thermo Scientific) at a constant flow rate of 250 nL/min at 40 °C. Peptide elution was achieved with a 150 min gradient from 1 to 50% of mobile phase B (A: 0.1% formic acid; B: 0.1% formic acid in acetonitrile). MS analysis was carried out in two steps, first acquisition of MS between 200 and 2000 m/z followed by Higher-energy collisional dissociation (HCD) of the 12 highest ions from each segment with normalized collision energy (NCE) 25, 30 and 35, using a dynamic exclusion duration of 30 s^90^.

### LC–MS/MS data analysis

LC–MS/MS data analysis was performed using PatternLab for proteomics 4.0 software (http://www.patternlabforproteomics.org) data analysis protocol^91^. The proteome from *Mus musculus* was used to identify the proteins present in the media. A target-reverse database including the most common contaminants was generated using PatternLab’s database generation tool. Thermo raw files were searched against the database with the following parameters: mass tolerance from the measured precursor m/z (ppm): 40; enzyme: trypsin; enzyme specificity: fully-specific; missed cleavages: 2; variable modifications: methionine oxidation; fixed modifications: carbamidomethylation of cysteine. Peptide spectrum matches were filtered using PatternLab’s Search Engine Processor (SEPro) module to achieve a list of identifications with less than 1% of false discovery rate (FDR) at the protein level^92^.

PatternLab’s Venn diagram module was used for pinpointing proteins exclusively identified in one biological condition using a probability mode (P < 0.05) following a Bayesian model. PatternLab’s TFold module was used to identify proteins found in both conditions but having a statistically differential abundance according to their extracted ion chromatograms (XIC)^93^. TFold module relies on the Benjamini–Hochberg theoretical FDR estimator to maximize the number of identifications that satisfy both a fold-change cutoff that varies with the t-test P-value as a power law and a stringency criterion that aims to filter out lowly abundant proteins that could produce false positives. Proteins identified in at least four of the six biological replicates were considered^90^. Hierarchical clustering was performed using the PatternLab module Clustergram, and Principal component analysis (PCA) was performed with the PatternLab module Buzios.

### Pathway enrichment analysis

Pathway enrichment analysis (also known as over-representation analysis) was performed in the Enrichr program^94,95^ with the Reactome pathway database (https://reactome.org/)^96^. The genes which coded for each of the proteins identified either exclusively or significantly increased in one condition, were assigned with the Uniprot database^97^.

The percentage of proteins in each condition that had been previously found in the extracellular compartment was determined using the “Annotation Tool” of the Database for Annotation, Visualization and Integrated Discovery (DAVID) Bioinformatics Resources v6.8^98,99^. Proteins assigned to the terms “extracellular region”, “extracellular space”, “extracellular matrix”, “extracellular vesicle”, “secretory vesicle” of the Gene Ontology Cellular Component database^100^ were considered extracellular proteins. Compartment analysis was also performed with the Enrichr program^94,95^ and the Jensen compartment database^101^.

### Gene expression analysis

Total RNA was extracted with TRIzol reagent (Thermo Fisher Scientific) and precipitated with isopropanol. The isolated RNA was quantified at A_260nm_ and the purity of the sample was controlled measuring A_260_/A_280_ ratio in a NanoDrop (Thermo Fisher Scientific). Prior to cDNA synthesis, 1 µg of total RNA was treated with DNAse-I (Thermo Fisher Scientific). Retrotranscription was performed in a final volume of 20 µl in the presence of random primers (200 ng), dNTPs (0.5 mM), DTT (0.01 M), RNaseOUT (40 U) and M-MLV Reverse Transcriptase (Invitrogen).

Quantitative RT-PCRs were carried out using QuantiTect® SYBR® Green PCR Kit (Qiagen) with specific primers in a Rotor-Gene 6000 analyzer (Corbett). Beta-Actin encoding gene *Actb* was used as house-keeping gene. The relative mRNA amount in each sample was calculated using the 2^−ΔΔCt^ method where ΔCt = Ct_gene of interest_ − Ct_Actb_^102^. Results were expressed as relative fold change in mRNA levels compared to control cells.

Primer sequences were as follows:

**Table:** 

Gene (protein or rRNA)	Forward 5′–3′	Reverse 5′–3′
*Mfn1* (mitofusin 1)	CCAGGTACAGATGTCACCACAG	TTGGAGAGCCGCTCATTCACCT
*Lgals9* (galectin-9)	TCAAGGTGATGGTGAACAAGAAA	GATGGTGTCCACGAGGTGGTA
*Cdkn1a* (p21)	TCCAGACATTCAGAGCCACAGG	ACTTTGCTCCTGTGCGGAAC
*Ccl5* (CCL5)	CCACCTCCTCTCTGGGTTGG	GTGCCCACGTCAAGGAGTAT
*Actb* (β-actin)	GCTTCTTTGCAGCTCCTTCGT	CGTCATCCATGGCGAACTG
*Ccl2* (C–C motif chemokine 2 / MCP-1)	ATTGGGATCATCTTGCTGGT	CCTGCTGTTCACAGTTGCC
*Tgfb* (TGF-beta-1)	GGAAATTGAGGGCTTTCGCC	CCGGTAGTGAACCCGTTGAT
*Il6* (IL-6)	GTTCTCTGGGAAATCGTGGAAA	AAGTGCATCATCGTTGTTCATACA
*Mtnd4* (NADH-ubiquinone oxidoreductase chain 4)	TCGCCTACTCCTCAGTTAGC	ATGTGAGGCCATGTGCGATTA
*Mtnd5* (NADH-ubiquinone oxidoreductase chain 5)	GCTTATCCTCACCTCAGCCAAC	ATTTGCGTCTGTTCGTCCGTA
*Mt-Rnr1* (12S rRNA)	ACCGCGGTCATACGATTAAC	CCCAGTTTGGGTCTTAGCTG
*Mt-Cyb* (Cytochrome b)	CTTAACCTGAATTGGGGGCCA	GTATGAGATGGAGGCTAGTTGG
*Mtatp6* (ATP synthase subunit a)	GCAGTCCGGCTTACAGCTAA	GGTAGCTGTTGGTGGGCTAA
*Mtatp8* (ATP synthase protein 8)	CATCACAAACATTCCCACTGGC	TCTCAAGGGGTTTTTACTTTTATGG
*Mtco1* (Cytochrome c oxidase subunit 1)	TCTGTTCTGATTCTTTGGGCAC	GGTGGGCTCATACAATAAAGCCT
*Mt-Co3* (Cytochrome c oxidase subunit 3)	CTTTGCAGGATTCTTCTGAGCG	GTCAGCAGCCTCCTAGATCAT
*Mfn2* (mitofusin 2)	TCCAAGGTCAGGGGTATCAG	GCAGAACTTTGTCCCAGAGC

### Assessment of mtDNA damage

Total DNA was extracted from cultured cells with Quick-DNA^TM^ Miniprep Plus Kit from Zymo Research following the manufacturer’s instructions. DNA was determined on a Qubit 4 Fluorometer (Invitrogen) using the dsDNA BR Kit (Invitrogen). Analysis of mtDNA damage was performed by real-time PCR on ECO™ (PCRmax). The reaction mixture included 2× SensiFAST SYBR No-ROX KIT (meridian BIOSCIENCE), forward and reverse primers (0.5 µM) and 10 ng of DNA. Primers targeting the D-loop region of mice mtDNA were used as described^25,103^: 5′-AAGAAGGAGCTACTCCCCACC-3′ (common forward for the short and long fragments); 5′-AGCTTATATTGCTTGGGGAAAATAGT-3′ (reverse for the short fragment); 5′-GTTGACACGTTTTACGCCGA-3′ (reverse for the long fragment). PCR cycling: 10 min at 95 °C, 35 cycles of 10 s at 95 °C, 30 s at 61 °C, 2 min at 72 °C. The number of lesions per 10 Kb was calculated a according to^25^:

$${\text{Number of lesions}}/{1}0\,\,{\text{Kb }} = ({1} - {2}^{{ - (}{\Delta {\text{Ct treatment }} - \Delta {\text{Ct control}})}} ) \, \times \, ({1}0,000/{\text{fragment length }}\left[ {{13}0{8}\,{\text{bp}}} \right]).$$

Cells exposed to 500 µM H_2_O_2_ for 30 min were included as a positive control for mtDNA damage, as described^25,26^. The sizes of qPCR products were verified by electrophoresis in agarose gels.

### mtDNA/nDNA ratio

DNA was extracted as described above. Equal amounts of total isolated genomic DNA (10 ng) were used for amplification by real-time quantitative PCR with QuantiTect® SYBR® Green PCR Kit (Qiagen) and 0.5 µM of each primer in a Rotor-Gene 6000 analyzer (Corbett). Separate tubes were used for mtDNA and nDNA amplification. PCR primer sequences for murine mitochondrial encoded gene 12S rRNA: 5′-ACCGCGGTCATACGATTAAC-3′ (forward) and 5′-CCCAGTTTGGGTCTTAGCTG-3′ (reverse); and nuclear encoded gene 18S rRNA: 5′-CGCGGTTCTATTTTGTTGGT-3′ (forward) and 5′-AGTCGGCATCGTTTATGGTC-3′ (reverse) were used^104^. The mtDNA/nDNA ratio was calculated as 2^(Ctn−Ctmt)^, assuming a doubling of DNA copy per cycle, and results were expressed as fold change relative to control condition^104^.

### Immunofluorescence

Cells were cultured in glass slides or coverslips, fixed in 4% paraformaldehyde, permeabilized with 0.1% Triton in PBS, incubated in blocking buffer (3% BSA, 0.1% Triton in PBS) for 1 h and incubated with a primary antibody anti-MFN1 (ab104274 Abcam 1:100) overnight at 4 °C and a secondary antibody Alexa Fluor® 488 goat anti-rabbit IgG (H + L) (#A11034, Thermo Fisher Scientific). 4′6-diamidino-2-phenylindole (DAPI, 1 μg/ml, Thermo Fisher Scientific) was used to stain the nuclei. Images were obtained by epifluorescence microscopy (Nikon Eclipse TE200).

### Growth and viability assays

Cells in culture were detached by incubation with Trypsin–EDTA (0.05%, 0.02%), and the number of viable and dead cells determined after staining with Trypan blue and counting in a Neubauer camera^105^. In cell growth assays cultured cells were treated with DMSO or TMZ as described before, and the number of viable cells was counted at different time points.

In the viability assays cells were exposed to the same conditions as described above for the collection of conditioned media for proteomic analysis both dead and live cells were considered. To determine the sensitivity of cell lines to TMZ 5 × 10^4^ cells were seeded in 24-well plates and treated with 50, 100, 150, 200 μM of TMZ. After 24 h cells were detached and viability determined as described above. The IC50s were calculated with GraphPad Prism.

### Wound healing

Cells were cultured in 6-well plates until confluency, a scratch was made using a cell scraper through the cell monolayer. Cells were imaged by light microscopy (Nikon Eclipse TE200) at 0, 8 and 24 h after scratch. Wound area was determined using the protocol from ImageJ wound healing plugin^106,107^.

### Lactate dehydrogenase activity

Cells were exposed to the same conditions as described above for the collection of conditioned media for proteomic analysis. The medium was collected, centrifuged at 10,000*g* to remove cells debris and used to perform the Lactate Dehydrogenase (LDH) Assay (Kit cat# E-107, Biomedical research service center) according to the manufacturers’ indications. The LDH assay is based on the reduction of a tetrazolium salt to formazan, in a NADH-coupled enzymatic reaction. Spectrophotometric measurements were performed at 492 nm in a plate reader (Varioskan, Thermo Fisher).

### Immunoblotting

Cells were routinely lysed in cell lysis buffer containing 20 mM Tris–HCl (pH 7.5), 150 mM NaCl, 0.1% Triton X-100, 1 mM EDTA, 1 mM EGTA, 2.5 mM sodium pyrophosphate, 1 mM β-glycerophosphate, 1 mM Na_2_VO_4_, 1 mM NaF, 1 mM PMSF, supplemented with Sigma FAST protease inhibitor cocktail and Calbiochem phosphatase inhibitor cocktail. Lysates were sonicated, centrifuged at 10,000*g* for 10 min and stored at − 80 °C. Protein concentration was determined with bicinchoninic acid (Thermo Fisher Scientific). Proteins (20–40 µg) were resolved by SDS-PAGE and transferred to nitrocellulose membranes at 4 °C overnight using standard Western blotting procedures. Membranes were cut to the same size of the gel before incubating with primary antibodies (5–10 ml) overnight at 4 °C. Anti-α-tubulin (05-829 1:1000) was from Millipore and anti-Galectin-9 (ab129075, 1:1000) was from Abcam. Secondary antibodies: IRDye® 800 anti-Rabbit IgG (H + L) (#926-32211, 1:10,000) and IRDye® 680 anti-Mouse IgG (H + L) (#926-68070, 1:10,000) from LI-COR Bioscience were used and immunoblots were visualized with the Odyssey (LI-COR Biosciences) and analyzed with Image Studio Lite (LI-COR Bioscience), an exposure of 2–4 was used for all blots. The scanned area was fitted to the membrane edges. We avoided scanning zones with no membrane to prevent high contrast since it diminished the quality of the images. This is why membrane edges are not visible in most of the blots.

### ELISA

Cells were seeded in 6-well plates and three days after TMZ treatment cells were washed with PBS and added 1 ml of DMEM without FBS. Two days after the medium was collected, centrifuged at 10,000*g* to remove cells and debris and stored at − 80 °C. Galectin-9 was assessed by ELISA using mouse galectin- 9 ELISA Kit (ab277407, Abcam). Results were normalized considering protein content (μg) determined by the bicinchoninic acid assay.

### Animal model

Female C57BL/6 mice (Dilave, Uruguay), 6–8 weeks old and weighing 19–23 g, were used for in vivo experiments. Animals were housed on 12:12 h light/dark cycles with controlled temperature (22 ± 2 °C) and humidity (60%), with water and food ad libitum. All animal experimentation protocols were approved by the University’s Ethical Committee for Animal Experimentation (CHEA), Uruguay and were in accordance with current national laws on Animal Experimentation (protocols 1552 and 1793). This study was reported in accordance with ARRIVE guidelines 2.0^108^.

### Treatment of melanoma tumors with conditioned media.

C57BL/6 mice were subcutaneously inoculated in the right flank with 2.5 × 10^5^ melanoma B16-F1 cells, giving rise to only one primary tumor per mice. When tumors were palpable, at day 10 post-tumor inoculation (p.t.i.), mice were divided into four groups (n = 12 mice per group) and injected intratumorally, for 3 consecutive days, with conditioned media from shScr DMSO (control), shScr TMZ, shMfn1 DMSO or shMfn1 TMZ cells (100 μl). Conditioned media were obtained as described above. Mice were controlled every 2–3 days, tumor size was measured with a caliper and the volume was calculated as: length × width × depth × π/6. Overall survival was assessed considering the day that mice died. Mice were sacrificed by cervical dislocation when tumor volume exceeded 4000 mm^3^ or before if they showed any sign of distress.

### Treatment of melanoma tumors with DTIC

C57BL/6 mice were inoculated subcutaneously (2.5 × 10^5^ cells) or intradermically (1 × 10^5^ cells) with melanoma shScr or shMfn1 B16-F1 cells, in the right flank, giving rise to only one primary tumor per mice. When tumors were palpable (day 10–12 p.t.i.), half of the mice in each group were administered chemotherapy with dacarbazine (DTIC) 150 mg/kg/dose intraperitoneally for 3 consecutive days^48^. Consequently, mice were divided into four groups for their study (n = 10, mice per group): shScr (control), shScr DTIC (control treated with DTIC), shMfn1 (lacking mitofusin 1), shMfn1 DTIC (lacking mitofusin 1 and treated with DTIC). Tumor size was measured as described above and overall survival was assessed considering the day that mice died. Mice were sacrificed by cervical dislocation when tumor volume exceeded 4000 mm^3^ for subcutaneous tumors and 1000 mm^3^ for intradermal tumors, or before if they showed any sign of distress.

### Immune cell recruitment to the tumor

Mice were inoculated subcutaneously and chemotherapy applied as described above. Animals were sacrificed at day 25 p.t.i. and tumors were removed and prepared to obtain single-cell suspensions. Cells were immunostained at 4 °C in the dark for 30 min with the following antibodies CD3-PerCp-Cy5.5 (clone 17A2), CD8-PeCy7 (clone 53–6.7), CD11c-PE-Cy7 (clone HL3), CD45-FITC (clone IM7), CD45-PE (clone 30-F11), CD49b-PE (clone DX5), and MHCII Ab-FITC (clone AFG-120.1) from BD Pharmingen, (San Diego, CA, USA). CD4-APCCy7 (clone 6K1.5), CD206 (clone C068C2) and F4/80-PB (clone BM8) from Biolegend (San Diego, CA, USA). Flow cytometry data were acquired on a FACS Canto II cytometer and analyzed using FACS Diva software (BD Biosciences, Oxford, UK). Cytometry analysis was performed acquiring 200,000 events and gating on total cells, followed by a gate on single cells and then on CD45 + immune cells. NK cells and T lymphocytes were determined as CD3− CD49b + and CD3 + CD49b−, respectively, within CD45 + cell population. Then CD4 + and CD8 + T lymphocytes were determined as CD8− CD4 + and CD8 + CD4−, respectively, within CD3 + T cell population. Macrophages were defined as F4/80 + cells, within CD45 + immune cells; within this population M1 and M2 macrophages were defined as CD206- and CD206 + cells, respectively. Then dendritic cells were identified as CD11c + MHCII +, within NOT F4/80 + cell population. Percentages of tumor-infiltrating cells were expressed in relation to single cell population, to normalize tumor sizes.

### Statistical analysis and graphs

All plots and statistical analysis were performed using GraphPad Prism (GraphPad Prism Inc., La Jolla, USA). Statistical tests used an alpha of 0.05. Normality was tested using the Shapiro–Wilk test. The data were analyzed by two-tailed unpaired Student´s t test when comparing two groups; or one- or two-way ANOVA with Tukey post-hoc tests, as appropriate, when comparing more than two groups. Outliers were identified with the ROUT method (Q1%) and removed. Differences in survival times were determined using the long-rank (Mantel-Cox) test. In all cases P < 0.05 was considered statistically significant. Results show the mean ± S.D, and n values represent the number of independent biological samples (wells, culture dishes or animals per group/condition). All experiments and statistical analysis were performed with n ≥ 3.

### Supplementary Information

Supplementary Figures.

Supplementary Dataset S1.

Supplementary Dataset S2A.

Supplementary Dataset S2B.

Supplementary Dataset S3A.

Supplementary Dataset S3B.

Supplementary Dataset S4A.

Supplementary Dataset S4B.

Supplementary Dataset S5A.

Supplementary Dataset S5B.

## Data Availability

The mass spectrometry proteomics data have been deposited to the ProteomeXchange Consortium via the PRIDE^109^ partner repository with the dataset identifier PXD043779. The rest of the data generated during this study are included in the published article and the supplementary information.
