# Supplementary material for: Mitofusin 1 silencing decreases the senescent associated secretory phenotype, promotes immune cell recruitment and delays melanoma tumor growth after chemotherapy
Source: Sci Rep. 2024 Jan 9;14:909. doi: 10.1038/s41598-024-51427-7 (PMC10776601; doi:10.1038/s41598-024-51427-7)
Supplement: Supplementary file 1 — Supplementary Figures. [file 41598_2024_51427_MOESM1_ESM.pdf]

Mitofusin 1 silencing decreases the senescent associated secretory phenotype, promotes immune cell recruitment and delays melanoma tumor growth after chemotherapy

Doménica Tarallo[a], Jennyfer Martínez[a], Alejandro Leyva[b], Amy Mónaco[c], Carolina Perroni[d], Marcos Tassano[d], Juan Pablo Gambini[e], Mónica Cappetta[f] Rosario Durán[b], María Moreno[c*], Celia Quijano[a]*.

[a]Departamento de Bioquímica, Facultad de Medicina, and Centro de Investigaciones Biomédicas (CEINBIO), Universidad de la República, Uruguay.

[b] Institut Pasteur de Montevideo and Instituto de Investigaciones Biológicas Clemente Estable (IIBCE), Uruguay.

[c]Departamento de Desarrollo Biotecnológico, Instituto de Higiene, Facultad de Medicina, Universidad de la República, Uruguay.

[d]Area Radiofarmacia, Centro de Investigaciones Nucleares, Facultad de Ciencias, Universidad de la República, Uruguay.

[e]Centro Uruguayo de Imagenología Molecular (CUDIM) and Centro de Medicina Nuclear (CMN), Hospital de Clínicas Dr. Manuel Quintela, Facultad de Medicina, Universidad de la República, Uruguay.

[f]Departamento de Genética, Facultad de Medicina, Universidad de la República, Uruguay.

Supplementary figures

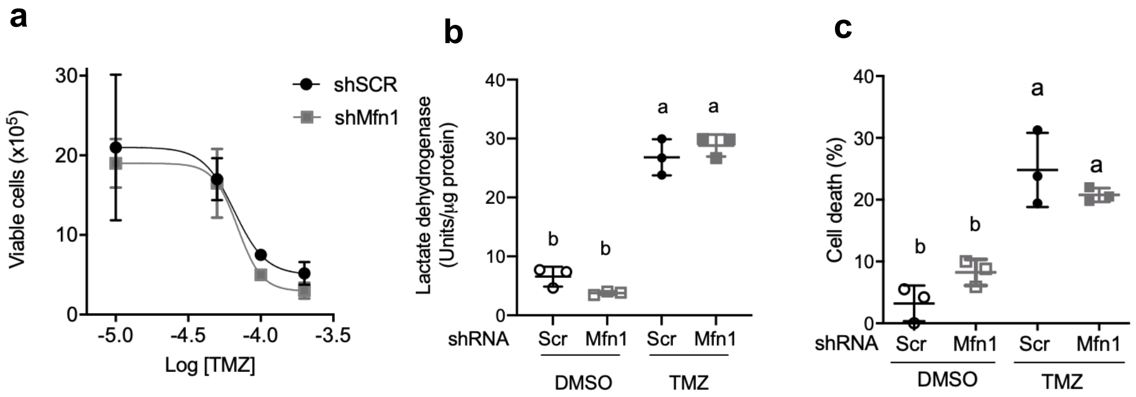

Supplementary figure S1: *Mfn1* silencing and treatment with TMZ in melanoma cells. B16-F1 cells were transduced with lentiviral particles carrying shScr or shMfn1 and selected with puromycin. shScr and shMfn1 cells were exposed to TMZ. **(a)** Cells were exposed to different concentrations of TMZ (50, 100, 150, 200 µM) for 5 h, after 24 h cells were detached and viability determined with Trypan blue. **(b)** Lactate dehydrogenase specific activity was measured in conditioned media obtained in the same conditions as for proteomic analysis. Two-way ANOVA, main effects of treatment ($P<0.0001$), shRNA ($P=0.74$) and their interaction ($P=0.07$). Tukey post-hoc for multiple comparisons, different letters are significantly different ($P<0.0001$). **(c)** Cell death was assessed with Trypan blue after incubation in the same conditions as for proteomic analysis. Two-way ANOVA, main effects of treatment ($P<0.0001$), shRNA ($P=0.8$) and their interaction ($P=0.06$). Tukey post-hoc for multiple comparisons, different letters are significantly different ($P\leq0.01$). The data are presented as the mean ± SD (n=3).

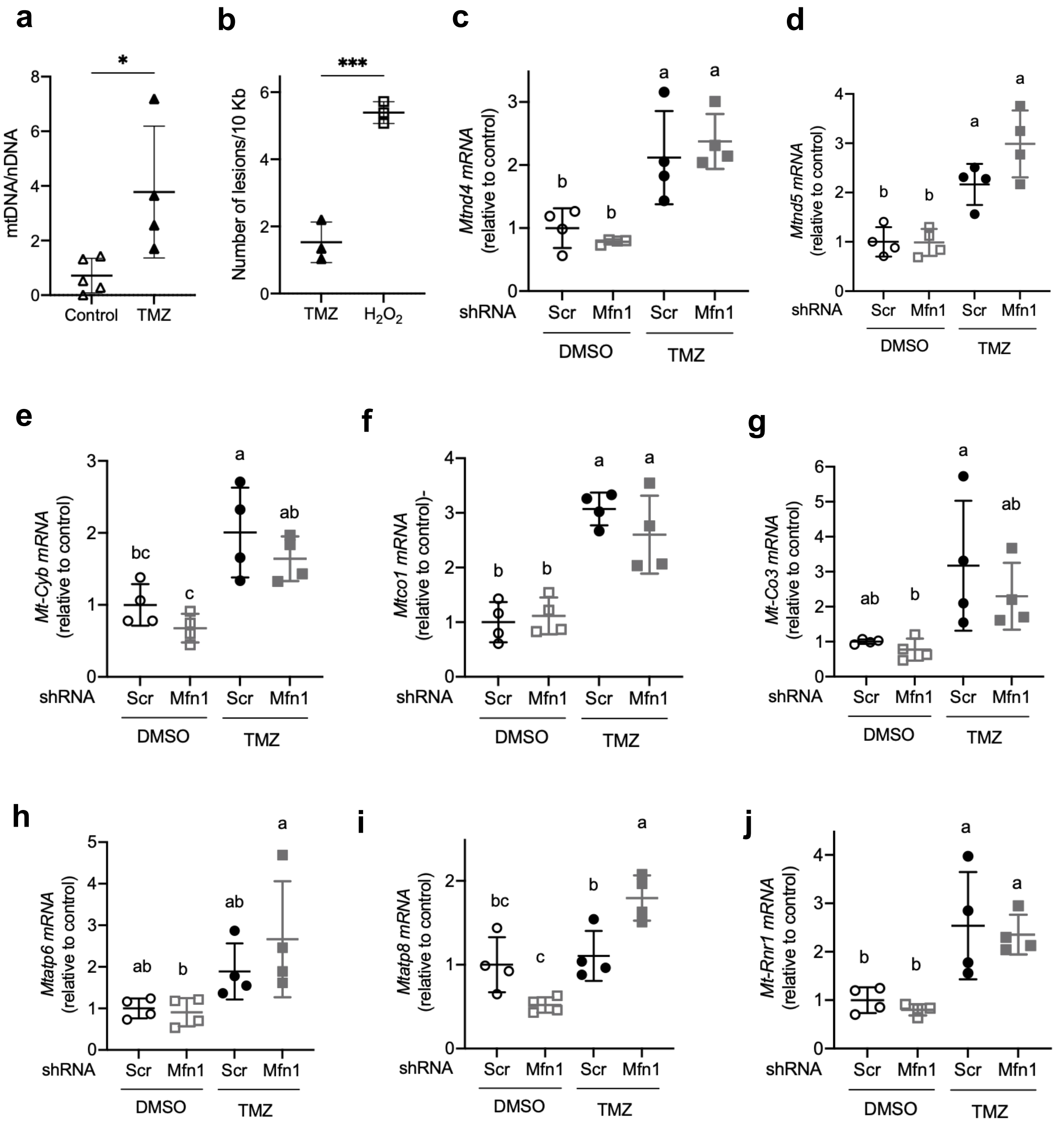

Supplementary Figure S2: mt-DNA damage, copy number and gene expression in senescent cells and non-senescent cell. Cells were exposed twice to TMZ (200 µM) or DMSO for five hours with a 24 h interval. Samples were analyzed five days after the last exposure to the drug. **(a)** mtDNA/nDNA ratio was determined by qPCR, unpaired t-test two-tails P=0.03. **(b)** DNA damage was determined by qPCR using primers for the D-loop, a positive control was generated exposing cells to 500 µM H_2O_2, unpaired t-test two-tails P=0.0006. **(c-j)** mRNA levels of genes encoded by mtDNA were assessed by RT-qPCR. Statistical tests: two-way ANOVA (main effects of treatment, shRNA and their interaction) and Tukey post-hoc for multiple comparisons, different letters are significantly different**:** **(c)** *Mtnd4* (P<0.0001, P=0.9 =, P =0.3), Tukey post-hoc P<0.02; **(d)** *Mtnd5* (P<0.0001, P=0.09, P=0.09), Tukey post-hoc P <0.01) **(e)** *Mt-Cyb* (P=0.0003, P=0.1, P=0.9), Tukey post-hoc P<0.02; **(f)** *Mtco1* (P<0.0001, P=0.2, P=0.5), Tukey post-hoc P<0.002; **(g)***Mt-Co3* (P=0.004, P=0.3, P=0.5), Tukey post-hoc P<0.03; **(h)** *Mtatp6* (P=0.006, P=0.4, P=0.3), Tukey post-hoc P<0.04; **(i)** *Mtatp8* (P=0.0002, P=0.4, P=0.0008), Tukey post-hoc P<0.03; **(j)** *Mt-Rnr1* (P =0.0003, P=0.5, P =0.9), Tukey post-hoc P<0.04. Results show the mean ± SD (n=3-4, per group).

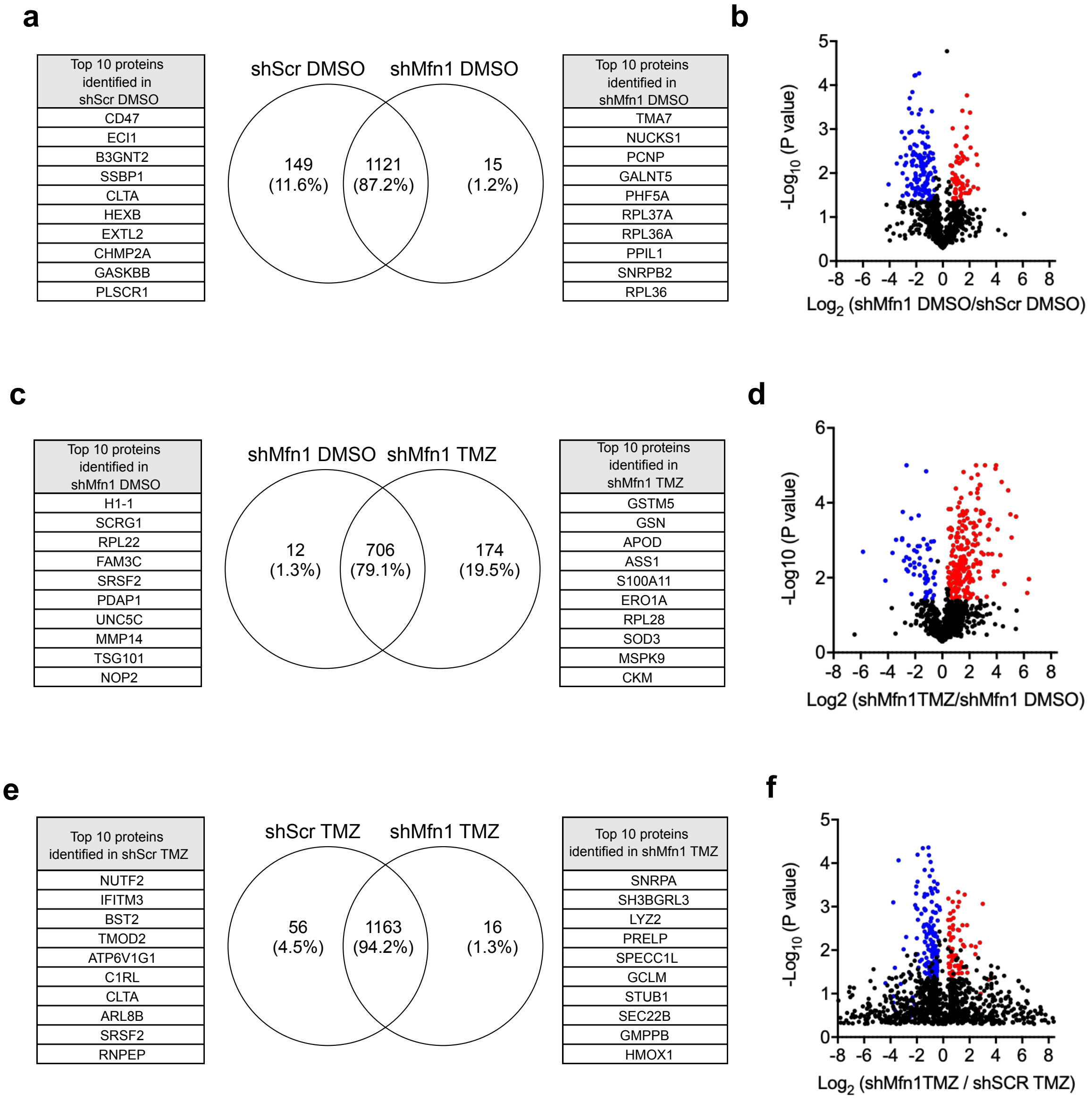

Supplementary Figure S3: Analysis of the secretome of melanoma cells after *Mfn1* silencing and treatment with a DNA damaging agent. Conditioned media were obtained from cells in the different conditions and pairwise quantitative analysis of the proteins identified using the shotgun approach was performed. **(a, c, e)** Venn diagrams showing proteins statistically exclusively detected in each condition (P<0.05). The tables show the 10 top proteins exclusively found in each condition (complete list in Supplementary Tables S2A, S4A and S5A). **(b, d, f)** Volcano plots showing in red or blue proteins with statistically differential abundance between conditions (q-value<0.05). Each dot represents a protein identified in at least four of the six biological replicates. Red and blue dots represent proteins significantly increased or decreased, respectively (the identity of each dot is shown in Supplementary Tables S2A, S4A and S5A). Differences were analyzed between: **(a)** non-senescent cells without or with mitofusin 1 (shMfn1 DMSO vs shScr DMSO), **(b)** senescent and non-senescent cells without mitofusin (shMfn1 TMZ vs shMfn1 DMSO), **(c)** senescent cells without or with mitofusin 1(shMfn1 TMZ vs shScr TMZ).

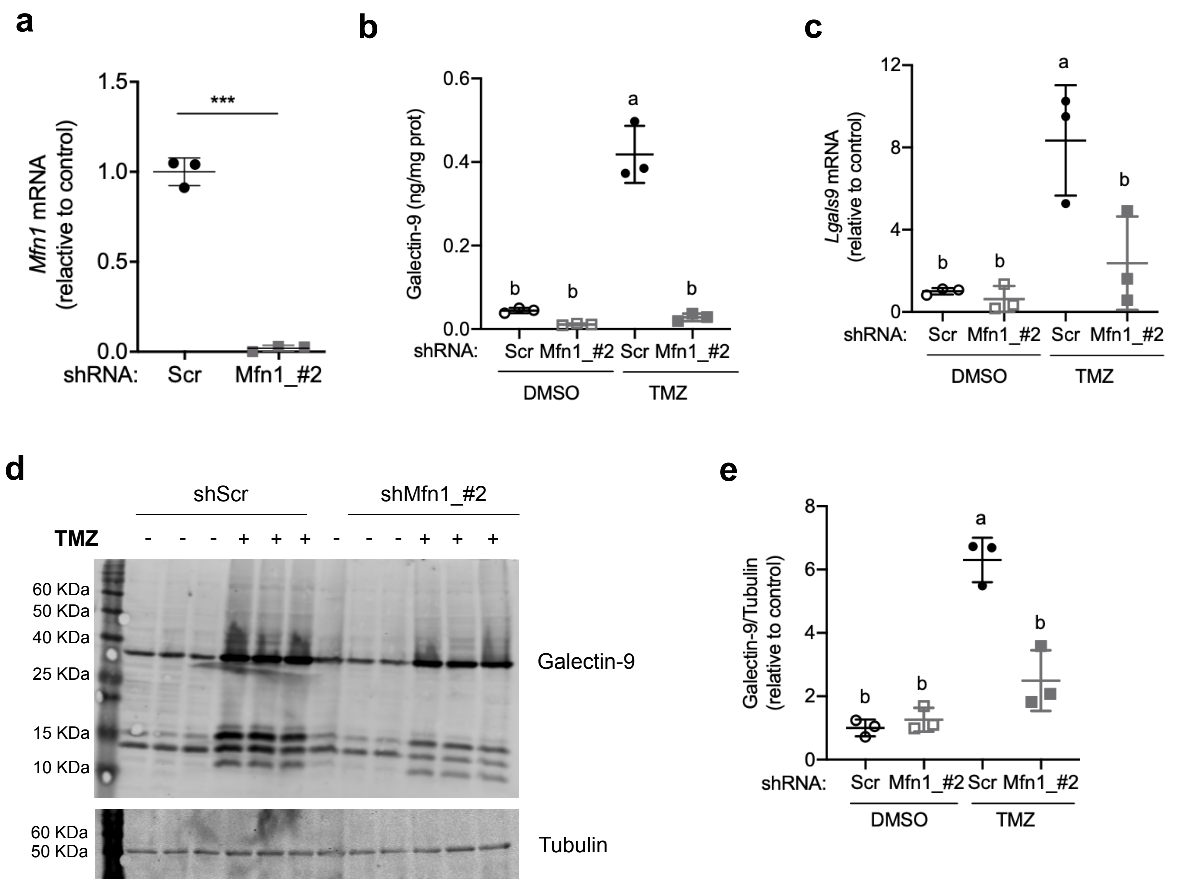

Supplementary Figure S4: *Mfn1* silencing with a second shRNA reduces Galectin-9 expression and secretion in senescent cells. B16-F1 cells were transduced with lentiviral particles carrying shScr or shMfn1_#2 (shRNA with a different sequence than shMfn1) and selected with puromycin. shScr and shMfn1 cells were exposed to TMZ. **(a)** *Mfn1* expression was determined by RT-qPCR and reported relative to control value. Unpaired t-test, two-tails, ***P<0.0001. **(b)** Galectin-9 secretion was assessed by ELISA. Two-way ANOVA, main effects of treatment (P<0.0001), shRNA (P<0.0001), and their interaction (P<0.0001). Tukey post-hoc for multiple comparisons, different letters are significantly different (P<0.0001). **(c)** *Lgals9* expression was determined by RT-qPCR, and reported relative to control value. Two-way ANOVA, main effects of treatment (P=0.002), shRNA (P=0.02) and their interaction (P=0.03). Tukey post-hoc for multiple comparisons, different letters are significantly different (P≤0.015). **(d)** Representative western-blot of intracellular levels of galectin-9. **(e)** Quantification of protein levels in (d). Galectin-9 was normalized using tubulin as loading control and results were expressed relative to control condition. Two-way ANOVA, main effects of treatment (P<0.0001), shRNA (P=0.0013) and their interaction (P=0.0006). Tukey post-hoc for multiple comparisons, different letters are significantly different (P≤0.0004). The data are presented as the mean ± SD (n=3). The uncropped images of the blots, with different degrees of exposure, can be found in Supplementary Figs. S10 and S11.

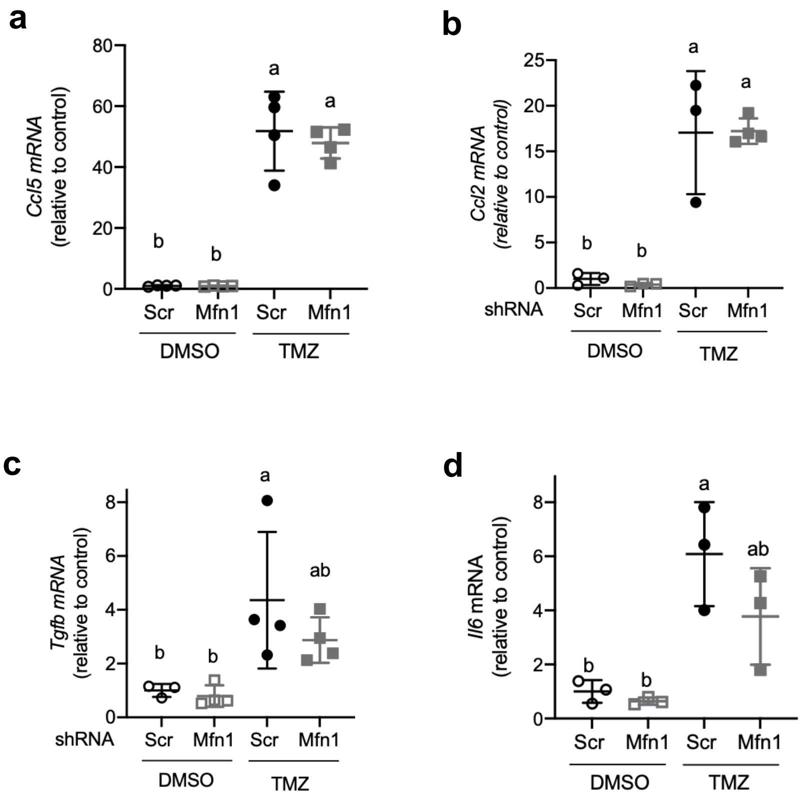

Figure S5: *Mfn1* silencing does not reduces the expression of several cytokines in senescent cells. ShScr and shMfn1 cells were exposed twice to TMZ (200 μM) or DMSO for five hours with a 24 h interval. Samples were analyzed five days after the last exposure to the drug. mRNA levels were assessed by RT-qPCR. Results show the mean ± SD (n=3-4, per group). Statistical tests: two-way ANOVA (main effects of treatment, shRNA and their interaction) and Tukey post-hoc for multiple comparisons, different letters are significantly different**:** **(a)** *Ccl5* (P<0.0001, P=0.6, P =0.6). Tukey post-hoc P<0.0001; **(b)** *Ccl2* (P<0.0001, P=0.9 =, P=0.8), Tukey post-hoc P=0.001; **(c)** *Tgfb* P=0.003, P=0.3 =, P=0.4), Tukey post-hoc P=0.02) **(d)** *Il6* (P=0.0007, P=0.1, P=0.2), Tukey post-hoc P=0.007.

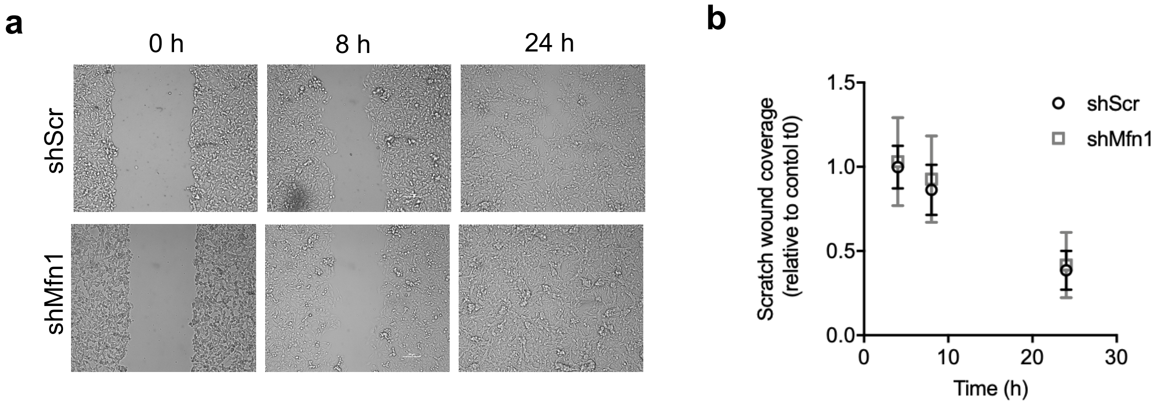

Supplementary Figure S6: *Mfn1* silencing does not affect melanoma cell migration assessed with the wound healing assay. ShScr and shMfn1 cells were seeded in culture plates and a scratch was made using a cell scraper through the cell monolayer. **(a)** Representative light microscope images to evaluate migration at 0, 8 and 24 h after scratch. **(b)** Wound area was determined using the protocol from ImageJ wound healing plugin. Results are the mean ± SD (n=6).

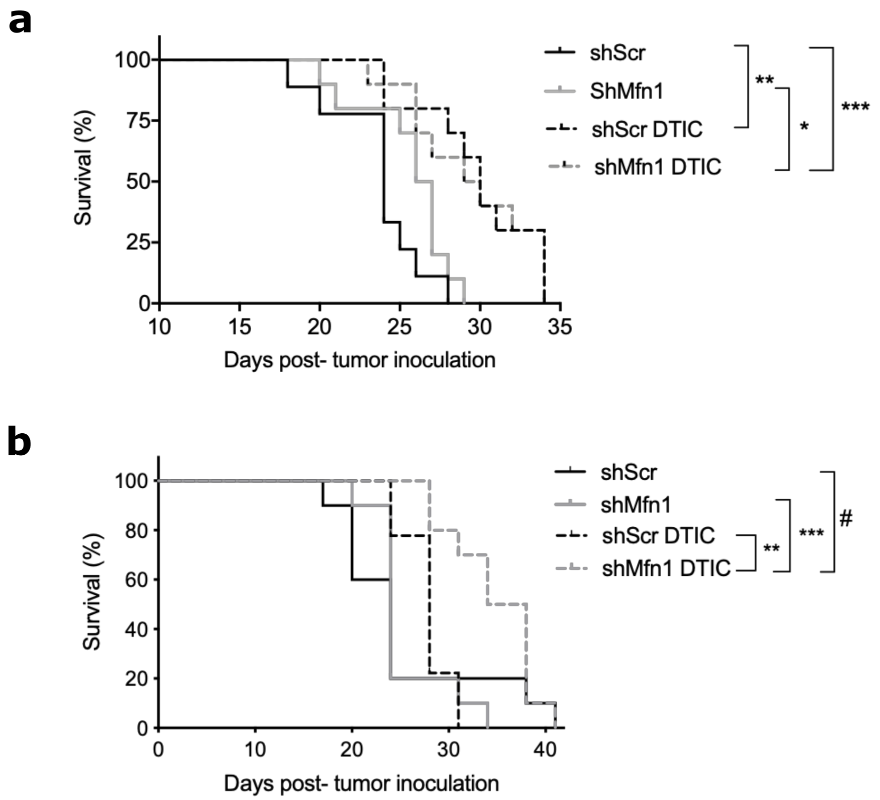

Supplementary Figure S7: ***Mfn1*** **silencing and survival of mice undergoing chemotherapy.** shScr or shMfn1 B16-F1 cells were injected subcutaneously or intradermically in the right hind leg of the mice. When tumors became palpable mice were treated with three doses of DTIC. **(a)** Survival after subcutaneous tumor cell inoculation. Log-rank Test *P=0.014, **P=0.002, ***P=0.001 (n=9-10). **(b)** Survival after intradermical tumor cell inoculation. Log-rank Test *P=0.02, **P=0.0009, ***P=0.0006, #P=0.05 (n=9-10).

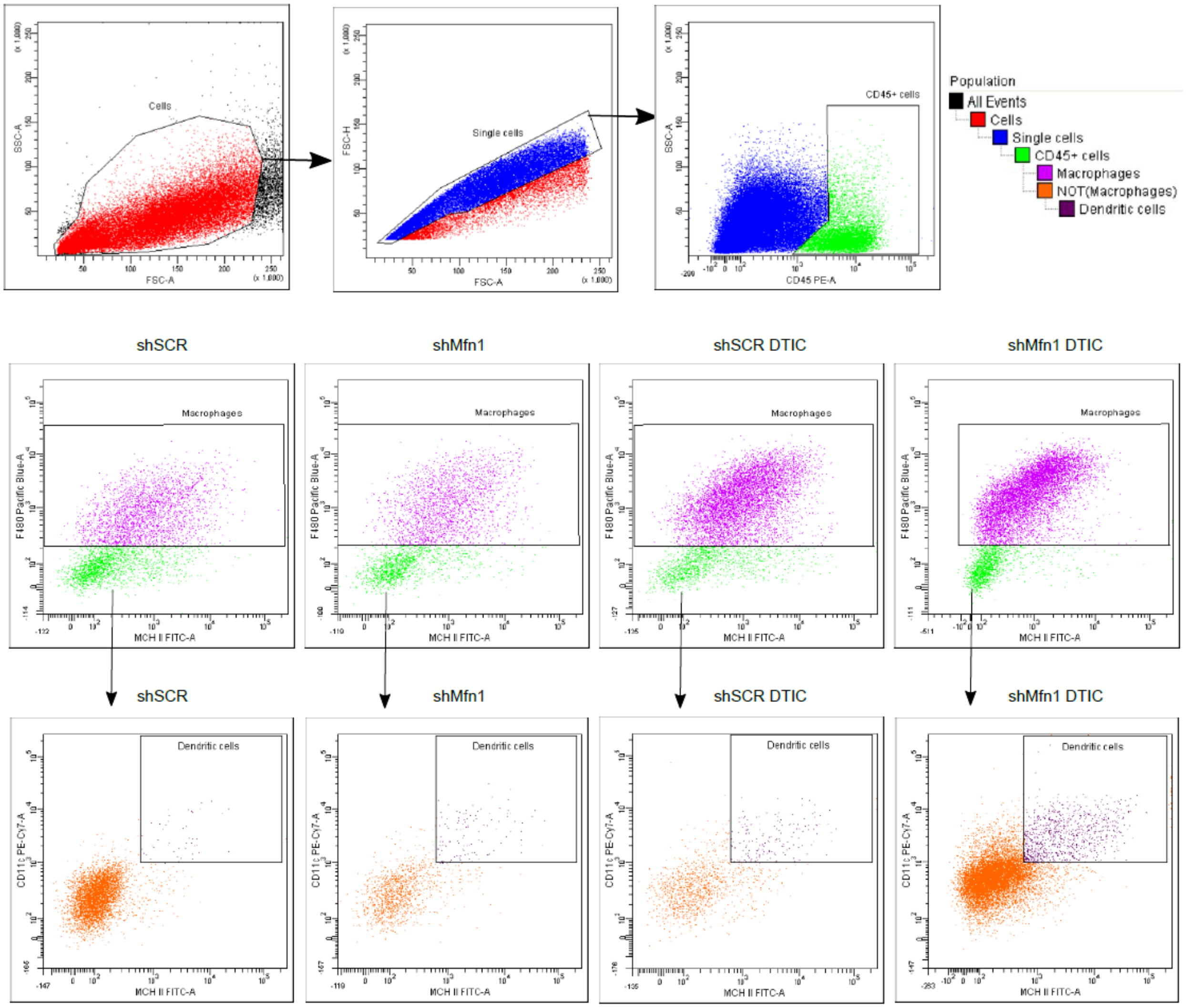

Supplementary Figure S8: Flow cytometry analysis of myeloid cells in the tumor. shScr or shMfn1 B16-F1 cells were injected subcutaneously in the right hind leg of the mice and when tumors became palpable (day 12 p.t.i.) mice were treated with three doses of DTIC. On day 25 p.t.i. tumors were excised and the percentages of tumor-infiltrating immune cells were analyzed as described in Methods**.** Flow cytometry analysis gating strategy and representative figures of macrophages and dendritic cells in the different conditions are shown. For analysis, 200,000 events were acquired. Gates were performed to eliminate cellular debris (FCS-A vs SSC-A) and doublets (FSC-A vs FSC-H). Cells were stained for CD45-PE, CD11c-PE-Cy7, MHCII A^b-FITC and F4/80-PB to defined immune cells (CD45$^+$), macrophages (CD45$^+$ F4/80$^+$) and dendritic cells (CD45$^+$ F4/80$^-$MHCII$^+$ CD11c$^+$).

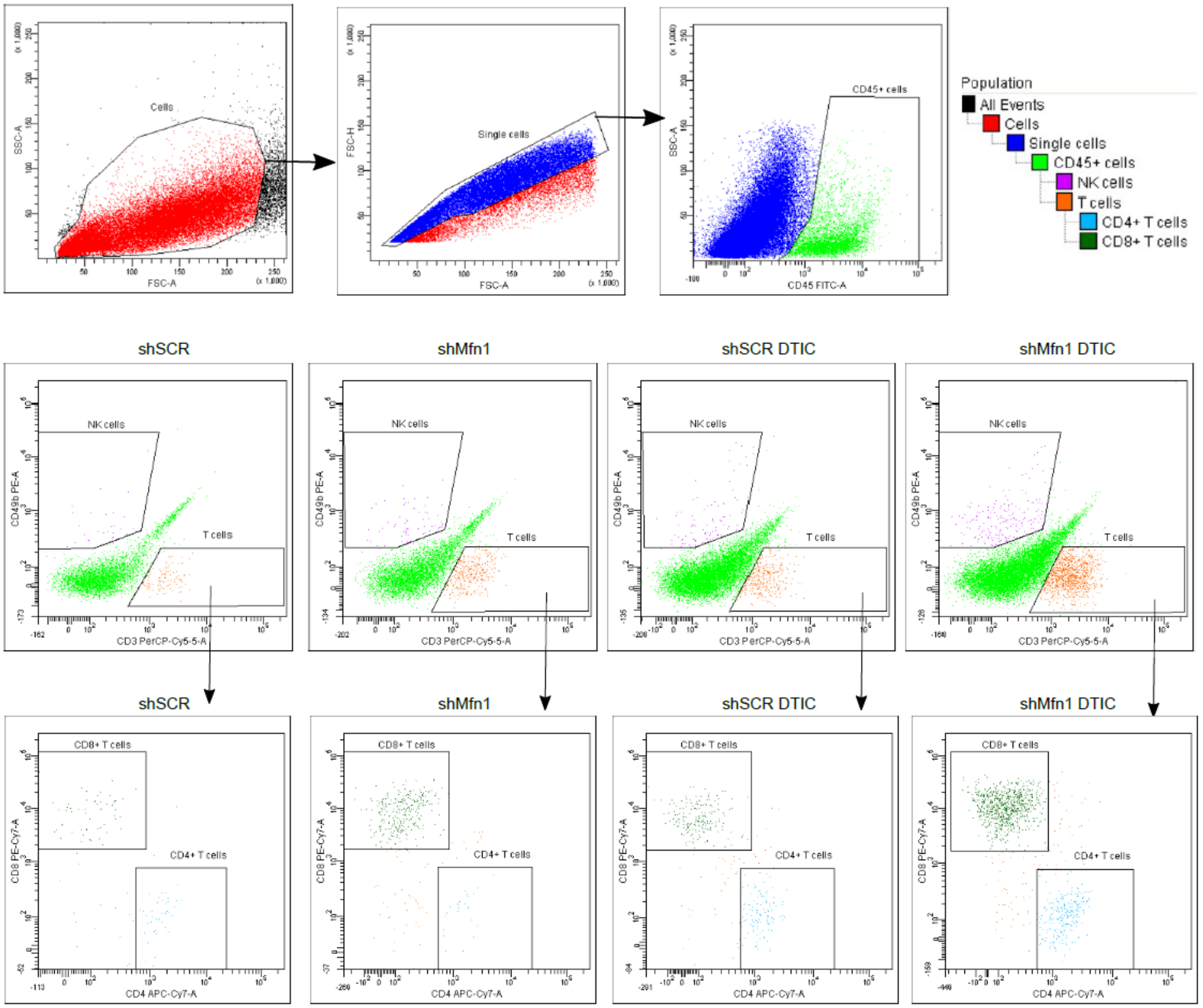

Supplementary Figure S9: Flow cytometry analysis of lymphoid cells in the tumor. shScr or shMfn1 B16-F1 cells were injected subcutaneously in the right hind leg of the mice and when tumors became palpable (day 12 p.t.i.) mice were treated with three doses of DTIC. On day 25 p.t.i. tumors were excised and the percentages of tumor-infiltrating immune cells were analyzed as described in Methods**.** Flow cytometry analysis gating strategy and representative figures of NK cells, T lymphocytes (CD8+ and CD4+) in the different conditions are shown. For analysis, 200,000 events were acquired. Gates were performed to eliminate cellular debris (FCS-A vs SSC-A) and doublets (FSC-A vs FSC-H). Cells were stained for CD45-FITC, CD3-PerCp-Cy5.5, CD8-PeCy7, CD49b-PE and CD4-APCCy7 to define immune cells (CD45$^+$), NK cells (CD45$^+$ CD49b$^+$ CD3$^-$), T cells (CD45$^+$ CD3$^+$ CD49b$^-$), CD8$^+$ T cells (CD45$^+$ CD3$^+$ CD49b$^-$ CD8$^+$ CD4$^-$), and CD4$^+$ T cells (CD45$^+$ CD3$^+$ CD49b$^-$ CD4$^+$ CD8$^-$).

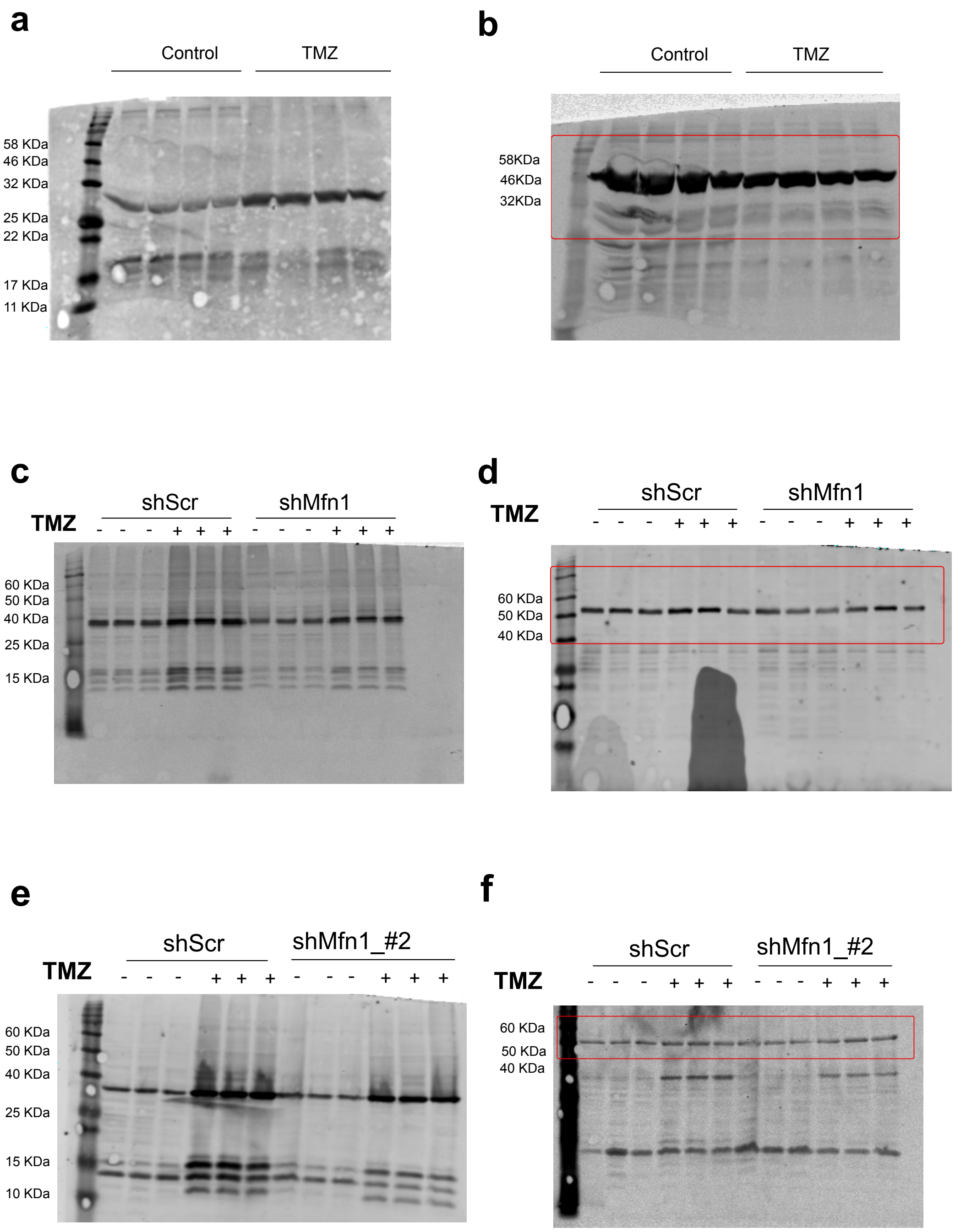

Supplementary Figure S10: Uncropped Western Blots. Uncropped western blots for the different figures: **(a)** Galectin-9 in Figure 3a. **(b)** Tubulin in Figure 3a. **(c)** Galectin-9 in Figure 3f. **(d)** Tubulin in Figure 3f. **(e)** Galectin-9 in Supplementary Figure S4d. **(f)** Tubulin in Supplementary Figure S4d. The area of the tubulin Western Blots that was cropped is shown in a red box.

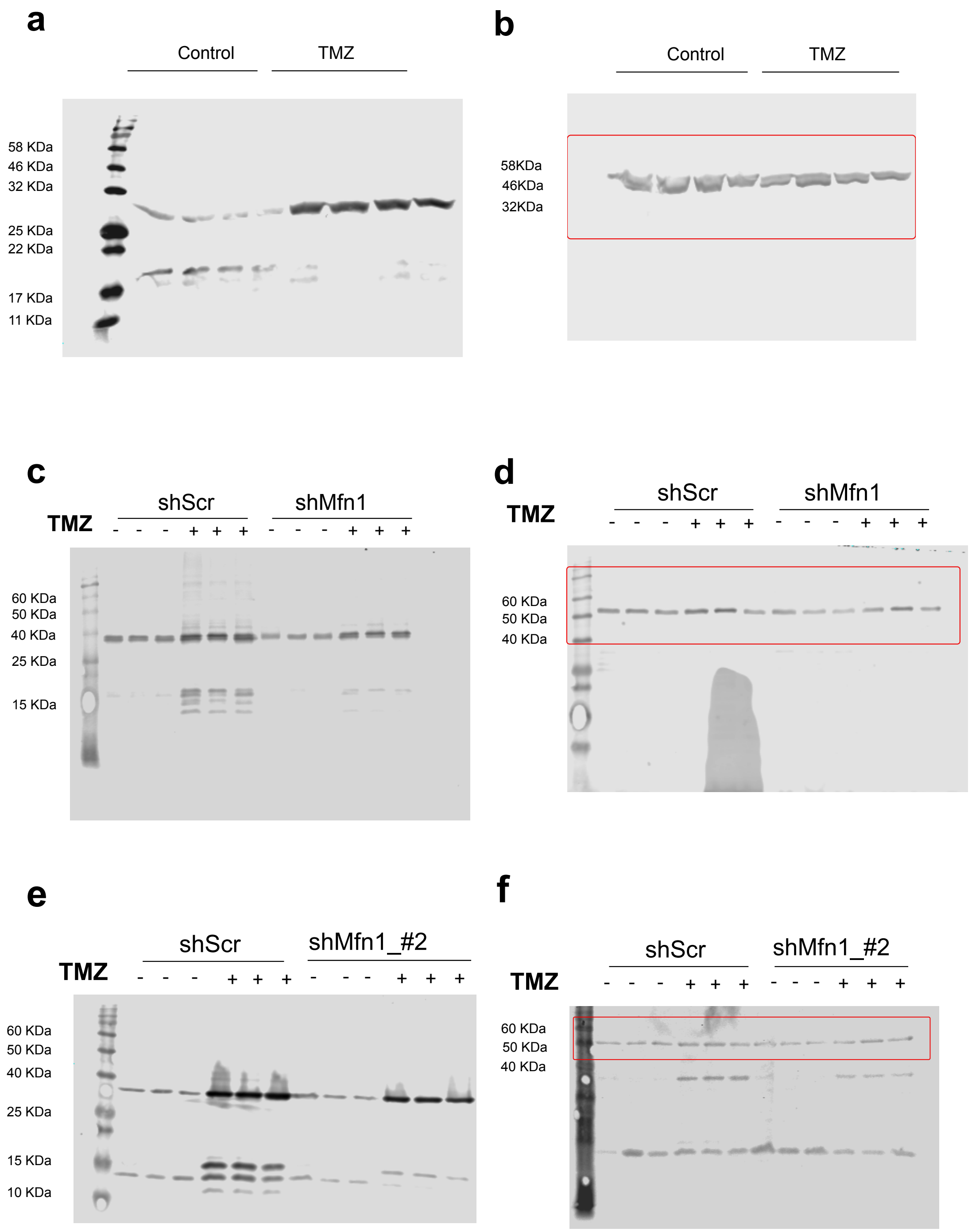

Supplementary Figure S11: Uncropped Western Blots with lower exposure. Uncropped western blots shown in Fig. S10 but with lower exposure, obtained with ImageJ: **(a)** Galectin-9 in Figure 3a. **(b)** Tubulin in Figure 3a. **(c)** Galectin-9 in Figure 3f. **(d)** Tubulin in Figure 3f. **(e)** Galectin-9 in Supplementary Figure S4d. **(f)** Tubulin in Supplementary Figure S4d. The area of the tubulin Western Blots that was cropped is shown in a red box.
